# Measurement of Sexual Interests with Pupillary Responses: A Meta-Analysis

**DOI:** 10.1007/s10508-021-02137-y

**Published:** 2021-09-23

**Authors:** Janice Attard-Johnson, Martin R. Vasilev, Caoilte Ó Ciardha, Markus Bindemann, Kelly M. Babchishin

**Affiliations:** 1 Department of Psychology, Faculty of Science and Technology, Bournemouth University, Dorset, BH12 5BB UK; 2 School of Psychology, University of Kent, Kent, UK; 3 Department of Psychology, Carleton University, Ottawa, ON Canada

**Keywords:** Sexual interest, Sexual arousal, Sexual orientation, Meta-analysis, Pupil dilation

## Abstract

Objective measures of sexual interest are important for research on human sexuality. There has been a resurgence in research examining pupil dilation as a potential index of sexual orientation. We carried out a meta-analytic review of studies published between 1965 and 2020 (*Mdn* year = 2016) measuring pupil responses to visual stimuli of adult men and women to assess sexual interest. Separate meta-analyses were performed for six sexual orientation categories. In the final analysis, 15 studies were included for heterosexual men (*N* = 550), 5 studies for gay men (*N* = 65), 4 studies for bisexual men (*N* = 124), 13 studies for heterosexual women (*N* = 403), and 3 studies for lesbian women (*N* = 132). Only heterosexual and gay men demonstrated discrimination in pupillary responses that was clearly in line with their sexual orientation, with greater pupil dilation to female and male stimuli, respectively. Bisexual men showed greater pupil dilation to male stimuli. Although heterosexual women exhibited larger pupils to male stimuli compared to female stimuli, the magnitude of the effect was small and non-significant. Finally, lesbian women displayed greater pupil dilation to male stimuli. Three methodological moderators were identified—the sexual explicitness of stimulus materials, the measurement technique of pupillary response, and inclusion of self-report measures of sexual interest. These meta-analyses are based on a limited number of studies and are therefore preliminary. However, the results suggest that pupillary measurement of sexual interest is promising for men and that standardization is essential to gain a better understanding of the validity of this measurement technique for sexual interest.

**Supplementary Information:**

The online version contains supplementary material available at 10.1007/s10508-021-02137-y.

## Introduction

The fact that the pupil of the human eye responds to changes in brightness levels within our visual environment is well known to most people. This is termed “the pupillary light reflex” and is responsible for an increase in pupil size (dilation) when the visual environment is darker and a decrease in pupil size (constriction) in brighter settings (Ellis, 1981). In the twentieth century, a number of incidental observations of pupils responding to factors other than light, including interest and arousal, led to the pivotal research of Löwenstein and Loewenfeld. They developed the first technological method for measuring pupils, the electronic pupillograph, which captured motion pictures of the pupil and recorded their fluctuating movements (Löwenstein & Loewenfeld, 1958). With this approach, it became possible to conduct experimental investigations into the physiological and psychological factors underlying pupillary responses (Loewenfeld, 1958; Löwenstein & Loewenfeld, 1962). Over the next decade, researchers began to investigate an array of mental activities linked to pupil size changes, including memory load (Kahneman & Beatty, 1966), and general arousal activation (Hess & Polt, 1960; Nunnally et al., 1967).

Fluctuations in pupil size are controlled by the autonomic nervous system and are a result of dynamic shifts between sympathetic (results in dilation) and parasympathetic (results in constriction) activation (McDougal & Gamlin, 2008; Zuckerman, 1971). A state of arousal leads to activation of the autonomic nervous system that is characterized by changes in skin conductance rate, blood pressure, breathing rate, and pupil dilation (Zuckerman, 1971). Pupil dilation can be evoked as a response to affective arousal, for example, during the viewing of highly pleasant (including sexually suggestive stimuli) and unpleasant stimuli (Bradley et al., 2008). Notably, the strength of the emotional state evoked by a stimulus (i.e., stimulus intensity) rather than the extent to which an emotion is positive or negative (i.e., stimulus valence) seems to elicit corresponding arousal responses in pupil size and change in skin conductance (Bradley et al., 2008). Sexually appetitive content, such as the viewing of erotic material or images depicting the observer’s arousing sex, has also been shown to evoke pupil dilation compared to neutral content or images depicting the non-arousing sex (Rieger & Savin-Williams, 2012). For example, pupillary response and skin conductance correlate positively during the viewing of erotic movies and pictures (see, e.g., Bernick et al., 1971; Finke et al., 2017). The close correlation between skin conductance and pupil size during arousal suggests that pupillary responses are predominantly driven by direct activation of the sympathetic nervous system. This may be different to pupil dilation observed during other cognitive processes, for example, during information-processing and mental load, which appear to be mediated by parasympathetic inhibition (Bradley et al., 2008; Steinhauer et al., 2004).

### Pupil Dilation as a Measure of Sexual Arousal/Interest

The ability to objectively measure sexual arousal and sexual orientation is important for research in understanding the theoretical basis of human sexuality. Existing measures of sexual orientation and sexual arousal include subjective self-report (e.g., Kinsey scale; Kinsey et al., 1948) and cognitive-based measures (e.g., IAT and Viewing Time; Babchishin et al., 2013; Lippa, 2012). However, these measures are recognized for their limitations; for example, subjective self-report would require the self-awareness of the individual as well as a willingness to respond truthfully, and there are debates around the precise mental processes that are being measured with cognitive-based tasks (Conaglen, 2004). Direct physiological measurement of sexual arousal is possible by recording genital responses (e.g., penile pleythsmography; Murphy et al., 2015). However, this is considered a highly invasive technique and may be of limited use for assessing individuals with sexual dysfunction. Pupillary responses could offer an additional or even alternative physiological measure of sexual arousal and sexual orientation to these measures, due to its non-invasive, instantaneous, and involuntary reaction to sexually appetitive content (Finke et al., 2017).

Hess et al. (1965) and Hess and Polt (1960) were the first to investigate whether changes in pupil size as a response to arousal are specific to preferred categories of pictorial sexual stimuli, which would therefore indicate subjects’ sexual orientation. The pupils of heterosexual and gay men were recorded with a camera sampling at a rate of two frames per second, while they viewed paintings and photographs of nude men and women. The pupils of all five heterosexual men dilated to the female pictures, while four of the five gay men experienced greater pupil dilation to male stimuli.

In the 50 years that followed publication of these findings, there were attempts to replicate these results. However, these studies reported contradictory findings. While Hess et al. (1965) demonstrated clear sex-specific pupillary responses, others failed to find differences in the pupillary responses of men and women (Aboyoun & Dabbs, 1998; Scott et al., 1967). These approaches varied widely in their methodology, which included marked differences in participant sex and sexual orientation, method for recording pupil size, variations in stimuli (e.g., nude versus dressed; photographs versus artistic illustrations; highly controlled versus natural), and analytical approaches to pupillary responses (Aboyoun & Dabbs, 1998; Hamel, 1974; Hess et al., 1965; Scott et al., 1967). These methodological differences, in combination with often elementary approaches for measuring pupil size, likely gave rise to the inconsistent response patterns recorded. Furthermore, the studies were often highly time-consuming, for example, requiring manual frame-by-frame measurement and limited data (e.g., recording only three pupil measurements per slide; Garrett et al., 1989). Consequently, during that time period fewer than ten attempts to replicate Hess et al.’s findings using comparable methodologies followed, with variable success (Bernick et al., 1971; Garrett et al., 1989; Hamel, 1974; Nunnally et al., 1967; Peavler & McLaughlin, 1967; Schnelle et al., 1974; Scott et al., 1967). As a result, the study of pupillometry for measuring sexual interests was largely abandoned.

A resurgence of this research occurred some 40 years after Hess et al.’s (1965) work with the increased accessibility of contemporary eye-tracking technology (see, e.g., Attard-Johnson & Bindemann, 2017; Attard-Johnson et al., 2016, 2017; Finke et al., 2017; Rieger & Savin-Williams, 2012; Rieger et al., 2015). This technology uses video-based pupil and corneal reflection for tracking and recording eye gaze position and pupil size with high precision, recording pupil size every 1 ms to 20 ms depending on equipment used. (A comparison of methodological technique is provided in greater detail in the “Measurement Techniques” section.) A number of findings emerged from these studies. For example, when observers view erotic video footage (e.g., male–male or male–female interactions, or solo male/solo female actions), pupil dilation occurs to content that is in line with their self-reported arousal and self-identified sexual orientation (Rieger & Savin-Williams, 2012). These response patterns have also been recorded during the viewing of static images depicting people in natural scenes (e.g., people wearing swim wear on beaches; Attard-Johnson et al., 2016, 2017), as well as for highly controlled sexually explicit and non-explicit images of adults (see Attard-Johnson & Bindemann, 2017). In these studies, for example, heterosexual men’s pupils are larger when viewing female adults. In contrast, gay men tend to show increased pupil sizes for same-sex stimuli. Furthermore, pupillary responses to sexual stimuli have also been shown to correspond with direct measures of genital arousal (Rieger et al., 2015) and with reaction time measures of sexual interest (Ó Ciardha et al., 2018), providing further validation for pupillary responses as a measure of sexual interest.

These studies show that pupillary responses are not only able to indicate an arousal response to sexual stimuli generally, but can change in response to categories of specific interest to an observer (Attard-Johnson & Bindemann, 2017; Attard-Johnson et al., 2016, 2017; Rieger & Savin-Williams, 2012; Rieger et al., 2015) and, as such, could provide an index of sexual orientation. However, recent studies of pupillary response with contemporary eye-tracking equipment also provide some contradictory results, indicating that a better understanding of this approach is still required. This variation in findings is reflected, for example, in sex differences between male and female observers.

### Sex Differences in Pupillary Response Patterns

Whereas men’s physiological measures of arousal correlate strongly with their self-reported sexual orientation and subjective appraisal of the stimuli being perceived, this relationship is much weaker in women (Bailey, 2009; Chivers et al., 2010). This inconsistent pattern of responding also seems to extend to the measurement of arousal with pupillary responses to sexually appetitive images (Attard-Johnson et al., 2016, 2017; Rieger & Savin-Williams, 2012; Rieger et al., 2015). In these studies, heterosexual women sometimes record larger pupil sizes for the other-sex consistent with sexual orientation (Attard-Johnson & Bindemann, 2017), sometimes indistinguishable pupillary responses for same and other-sex stimuli (Rieger et al., 2015), and sometimes larger pupil sizes when viewing same-sex stimuli (Attard-Johnson et al., 2016). While this variation in response patterns is evident in heterosexual women, lesbian women have a greater tendency to respond in concordance with their self-reported sexual orientation and subjective arousal ratings (Rieger & Savin-Williams, 2012).

A factor that could contribute to inconsistencies in female sexual responding might reflect differences in the stimuli that are used in different studies. Stimuli that have been employed in the measurement of female sexual response more broadly vary in terms of modality but are typically sexually explicit. All 132 studies published between 1969 and 2007 reporting genital and self-reported sexual arousal responses utilized stimuli depicting highly explicit sexual content comprising nudity, sexual acts, erotic video footage, erotic audio tapes, or sexual fantasy (studies reported in a meta-analysis by Chivers et al., 2010). Such stimuli have also been used extensively in more recent studies into measurement of genital responses in women (Dawson & Chivers, 2018). In contrast, research with pupillary response measurements differs not only in mode (e.g., static images versus video footage) but also in degree of sexual explicitness, from erotic videos depicting sexual acts (see Rieger & Savin-Williams, 2012; Rieger et al., 2015) to completely nude, semi-nude, and dressed images of men and women (see Attard-Johnson et al., 2016; Attard-Johnson & Bindemann, 2017). Although male sex-specific responding appears to be rather robust across these variations in stimuli, female responding is more varied (Attard-Johnson & Bindemann, 2017; Attard-Johnson et al., 2016; Finke et al., 2017; Rieger & Savin-Williams, 2012; Rieger et al., 2015).

These more varied responses in females could be a result of the preparation hypothesis (Dawson et al., 2013; Lalumière et al., 2020), which proposes that any sexual cues provoke automatic genital responses as a protective mechanism to prepare a woman for the possibility of a sexual encounter, and thus reduce the likelihood of pain or injury occurring (Chivers, 2017; Laan & Everaerd, 1995; Suschinsky & Lalumière, 2011). A time course of pupillary responses to explicit sexual stimuli reported in Finke et al. (2017) suggests that the pupils dilate indiscriminately to sexual stimuli within 400–600 ms of onset of image (when stimulus luminance is controlled for), followed by more specific responding. Specifically, early and late pupillary responses were associated with differential heart rate and skin conductance responses and suggest that these distinct responses are associated with different processes of the autonomic nervous system (Finke et al., 2017). Initial dilation (parasympathetically controlled), when not attributed to the light reflex, may be an immediate and spontaneous response to the detection of *any* biologically significant sexual cue. Later pupil dilation (sympathetically controlled) occurring after the initial 400 ms period may relate to stimulus content and sexual orientation (Finke et al., 2017). This can be seen in men where pupil size to all erotic stimuli (i.e., both other and same sex) started to differ significantly from baseline and from neutral non-erotic stimuli at 400 ms, whereas pupil size diverged after 600 ms for specific sexual stimuli according to sexual orientation (Finke et al., 2017). In women, however, the indiscriminate pupil responses between images of men and women continued throughout the duration of image presentation. The initial response to sexual cues provides some support for the preparation hypothesis and suggests that perhaps in women this response is sustained for longer and may be underpinned by a different biological process to that in men.

If the preparation hypotheses results in sustained responses in women, then images of high stimulus intensity depicting strong sexual cues, such as nude or erotic images of men and women, may trigger a heightened *non-specific* arousal response that attenuates any discrimination in arousal between image categories from women. If the sexual intensity of the images is reduced, for example, by viewing images of dressed men and women absent of all sexual cues, female responses may be less sensitive and become *more* discriminant across categories. Stimulus type and intensity are varied across studies measuring genital arousal, including audio-visual stimuli comparing arousal to human sex acts of varying intensity, primate sex acts, neutral scenes with no-person content (Chivers & Bailey, 2005), and audio narrative descriptions of sexual and non-sexual interactions (Chivers & Timmers, 2012). However, it is unclear from these studies whether the non-specific pattern of responding in women is a function of stimulus intensity. Furthermore, existing research measuring arousal with pupillary responses to non-sexually explicit stimuli of men and women is limited and also does not reveal a specific pattern. For example, while heterosexual women’s pupils were larger for the other-sex when viewing both nude and dressed stimuli of the same identity (Attard-Johnson & Bindemann, 2017), in a different paradigm dilation was more strongly correlated with sexual orientation when women viewed sexually explicit videos compared with non-explicit videos of men and women discussing the weather (Watts et al., 2016). Furthermore, other studies report pupil dilation consistent with sexual orientation when women were exposed to only the faces of men and women (Laeng & Falkenberg, 2007). It is therefore possible that the specific experimental stimuli of a study can affect the response patterns found in women. However, the precise mechanism and the extent to which this occurs are not clear and may be attributed to an interaction of multiple factors. One of these factors may be the technique that is applied to measure changes in pupil size.

### Measurement Techniques

Since the early studies, advancements in eye-tracking technology have made it easier to accurately measure pupil size changes. However, with limited research and no standardized approach, studies have varied widely in the methods of measurement and analysis (see Attard-Johnson et al., 2019), which could contribute to variations in pupillary response patterns across studies. The methods employed to measure pupillary responses differ particularly when comparing earlier measurement techniques, which were based on manual application of a ruler to calculate pupil size from still images of the eye, compared with modern techniques that apply automated infrared light to capture pupil responses.

One aspect in which these approaches vary relates to the rate of recording of pupil response, whereby early methods recorded far fewer pupil size measurements per stimuli compared to modern eye-tracking technology. Some earlier studies report only three recordings per trial using manual methods (Garrett et al., 1989) compared with a pupil measurement recorded every 1 to 20 ms for the duration of every trial with modern eye-tracking technology, such as the EyeLink (SR Research Ltd., 2005–2008), Tobii (2016), and SMI (SensiMotoric Instruments, Gaze Intelligence, 2020) eye trackers. Consequently, when scores for a trial are averaged across fewer pupil measurements, this could allow greater opportunity for erroneous pupil size changes unrelated to the task to have an undue influence on the overall task-evoked pupillary responses. Erroneous pupil dilation or constriction refers to noise in pupil dilation data arising from spontaneous internal cognitive processes unrelated to the task (e.g., working memory and cognitive load; Kahneman & Beatty, 1966), or even direction of eye gaze in relation to the stimulus. An additional advantage of modern eye trackers is that they can record the location of eye gaze alongside pupil responses. In this way, it is possible to eliminate erroneous pupil measurements that are linked to eye movements falling outside the boundaries of the stimulus or due to eye blinks.

Modern eye-tracking systems also differ from one another in their setup. For example, some eye trackers are desktop-mounted and involve the physical stabilization of the head using a chinrest to minimize head movements. Other remote eye trackers do not require physical head restraint but, instead, rely on computer algorithms to correct for head movements. The former generally provides greater accuracy while the latter allows more mobility (Brisson et al., 2013). There are several variations in the technological design, such as calibration quality and sampling rate, across a range of eye trackers that could provide a potential source of difference. The influence of these sources of error could be mitigated to some extent by reducing head movement with the use of a head or chin rest (Titz et al., 2018).

Environmental lighting is one factor that could provide a source of difference in accuracy between remote and desktop-mounted eye trackers. Although the effect of lighting has not been directly investigated in this context, one can imagine how lighting variation from the environment throughout an experiment can influence pupillary responses to the process being investigated. While researchers typically ensure consistent artificial lighting in the testing laboratory, remote eye trackers allowing for even minor head movements can pose a potential source of variation in light from the environment entering the pupil at different intensities. Subtle head movements, such as tilting the head backwards toward an overhead light or forwards and away from the light, can trigger a pupil light reflex (Ellis, 1981). Such changes in light intensity from the environment can cause pupil adaptation and recovery in the time following (Mathôt, 2018), which can risk interfering with the task-related pupil responses being measured. However, a systematic and direct comparison of pupil response measurement across different eye-tracking systems has not been performed and therefore differences in accuracy and reliability are yet to be established. Therefore, we cannot exclude the possibility that differences in eye-tracking methods could account for at least some of the discrepancies in findings across studies.

### Present Study

In recent years, there has been a marked increase in studies examining pupil responses as a method for assessing a person’s sexual interest in other adults. Available evidence, however, suggests that pupil responses are not a reliable measure across and within observer groups that differ in sex and sexual orientation. Furthermore, the existing studies diverge in their methodological approaches to using pupil responses to assess sexual interests. Consequently, uncertainty exists at present as to the sensitivity of pupillary responses as a measure of sexual interests and the impact of measurement approach on such effects. In this context, the present study provides a meta-analysis of existing research to examine the extent to which pupil responses provide a measure of sexual interests in adult men and women. For this purpose, difference scores comparing pupillary responses to images of males and females were calculated separately for men and women. This within-subjects approach was taken to examine the applicability of the method in assessing sexual interest among these groups. The meta-analysis also includes factors that may moderate the validity of this measurement technique. Specifically, moderator analyses were conducted to examine the extent to which stimulus sexual explicitness (completely nude, partially nude with sexual regions obscured, or completed dressed), pupillary response measurement technique, and self-report measures used to confirm sexual orientation were related to the sensitivity of pupillary response for assessing sexual interests.

Based on previous research, we expected that pupil sizes are consistently larger for stimuli consistent with sexual orientation in men (i.e., larger pupils for other-sex stimuli in heterosexual men, and for same-sex stimuli in gay men). For heterosexual women, we expected a weak pattern of pupillary responses that may be larger for male stimuli. In contrast, we expected that lesbian women show stronger pupil dilation to same-sex images. Bisexual men and women would be expected to show undistinguishable pupillary responses between male and female stimuli.

## Method

Studies available in peer-reviewed journals, books or book chapters, as well as unpublished dissertations, abstracts, conference papers, and preprints, were included in this review up until January 2020. Studies were identified by searching electronic research databases Web of Science and EBSCO (which includes PubMed, Medline, Science Direct, Open Dissertations, PsychINFO), by examining the reference lists of relevant studies, and via ResearchGate. Authors were contacted directly to request full texts, additional data, and information. The search terms employed were the following: pupil* (asterisk indicating variation such as pupillometry, pupil dilation, pupil responses, pupil size, pupillary) and sexual* (appeal, interest, arousal, preference, orientation), pupil* and attract* (attraction, attractive; see Figure S1 in Online Supplementary Materials).

**Fig. 1:**
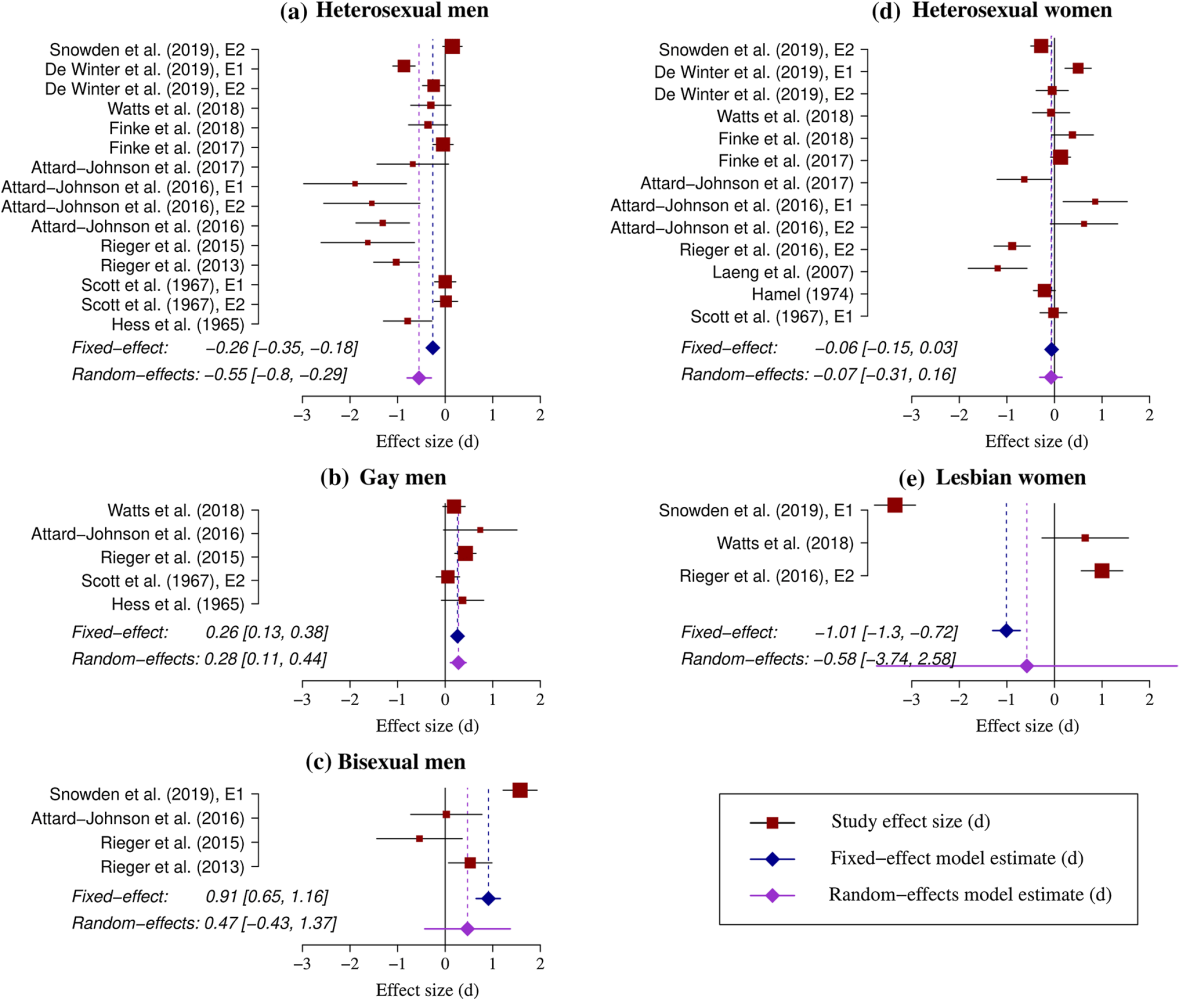
Forest plots for the main meta-analysis results for heterosexual men (**a**), gay men (**b**), bisexual men (**c**), heterosexual women (**d**), and lesbian women (**e**). Plotted are the effect size estimates for each study and the meta-analysis estimates (with 95% CIs). The size of squares is proportional to the fixed-effect weight of each study. Positive effect sizes indicate greater pupil dilation to same-sex stimuli, and negative effect sizes indicate greater pupil dilation to other-sex stimuli. Study No. 4 (Snowden et al., 2019, E1) was excluded as an outlier from the analysis in panels **a**, **b**, and **d**; study No. 24 (Rieger et al., 2013) was also excluded as an outlier in panel **b** (see the Online Supplementary Materials for more details)

To be included studies needed to meet the following criteria: They measured and compared pupil responses to visual stimuli of adult men and women to measure sexual interest; included at least one sample of male or female observers identifying as heterosexual, gay, or bisexual; stimuli (video or images) used depicted either a man or a woman but not both; a sample size of at least 5 observers per group; and contained sufficient information to calculate effect size *d*. In September 2019, the search yielded 40 documents following removal of duplicates. For studies reporting overlapping samples, the average effect size was taken if the sample size was identical, and the effect size for the study with the largest sample was taken if the sample size was not identical. In total, 16 eligible studies were identified (see Fig. 1). From the 17 studies, 16 included comparisons for heterosexual men, 7 for gay men, 4 for bisexual men, 14 for heterosexual women, 3 for lesbian women, and 2 for bisexual women. Before commencing analysis, another search was performed in January 2020 that revealed two additional studies for heterosexual men and women. The total number of studies eligible was increased to 19. See Table 1 for a summary of studies.

**Table 1:** Summary of descriptive information of eligible studies included in meta-analysis

Sample: Heterosexual men											
Study No.	Study	Country	*N*	Age	Setting	Sexual interest measure	Pupil response measure	Stimuli	Sexual explicitness	Duration per trial (ms)	Number of trials per category
1	Attard-Johnson et al. (2016)	UK	22	21.8	University/College	Self-report	EyeLink SR Research Remote infrared eye-tracker (500/1000 Hz sample rate recording every 2 ms)	Images—Photographs	Partial nudity (obscured sexual regions)	5000	5
2	Attard-Johnson et al. (2016)	UK	21	19.5	University/College	Self-report	EyeLink SR Research Remote infrared eye-tracker (500/1000 Hz sample rate recording every 2 ms)	Images—Photographs	Partial nudity (obscured sexual regions)	5000	5
4	Snowden et al. (2019)	UK	20	25.5	University/College/Community	Self-report (Kinsey Scale)	Tobii X2-60 Hz mobile eye-tracker (60 Hz rate recording every 16.67 ms)	Images—Photographs	Mixed nudity	2000	8
5	Snowden et al. (2019)	UK	18	21.6	University/College/Community	Self-report (Kinsey Scale)	Tobii X2-60 Hz mobile eye-tracker (60 Hz rate recording every 16.67 ms)	Images—Photographs	Mixed nudity	3000	10
7	Watts et al. (2018)	UK	6	25	University/College/Community	Self-report (Kinsey Scale)	EyeLink SR Research Remote infrared eye-tracker (500/1000 Hz sample rate recording every 2 ms)	Video footage	Complete nudity	18,000	3
9	Hess et al. (1965)	USA	5	29	University/College/Community	Self-report	Manual frame-by-frame measurement of pupil diameter from video footage	Images—Photographs and paintings	Mixed nudity	10,000	5
10	Finke et al. (2018)	Germany	17	24.1	University/College	Self-report	SMI iView-X HiSpeed 500 eye-tracker (500 Hz sample rate)	Images—Photographs	Complete nudity	2500	15
11	Finke et al. (2017)	Germany	14	25.3	University/College	Self-report	SMI iView-X HiSpeed 500 eye-tracker (500 Hz sample rate)	Images—Photographs	Mixed nudity	2500	48
14	Scott et al. (1967)	USA	10	–	University/College	Sexual interest not recorded	Manual frame-by-frame measurement of pupil diameter from video footage (2 f/s)	Images	Mixed nudity	10,000	8
15	Scott et al. (1967)	USA	5	26.5	Prison	Self-report	Manual frame-by-frame measurement of pupil diameter from video footage (2 f/s)	Images	Mixed nudity	10,000	8
17	Attard-Johnson et al. (2016)	UK	57	21.6	University/College	Self-report	EyeLink SR Research Remote infrared eye-tracker (500/1000 Hz sample rate recording every 2 ms)	Images—Photographs	Partial nudity (obscured sexual regions)	10,000	18
24	Rieger et al. (2013)	UK	94	28	University/College/Community	Self-report (Kinsey Scale)	EyeLink SR Research Remote infrared eye-tracker (500/1000 Hz sample rate recording every 2 ms)	Video footage	Complete nudity	30,000	1
29	Attard-Johnson et al. (2017)	UK	20	22.4	University/College	Self-report (Kinsey Scale)	EyeLink SR Research Remote infrared eye-tracker (500/1000 Hz sample rate recording every 2 ms)	Images—Photographs	Mixed nudity	2669 (average duration)	18
39	De Winter et al. (2021)	Netherlands	129	23.2	University/College	Sexual interest not recorded	EyeLink SR Research Remote infrared eye-tracker (500/1000 Hz sample rate recording every 2 ms)	Images—Photographs	Partial nudity (obscured sexual regions)	10,000	2
40	De Winter et al. (2021)	Netherlands	102	23.3	University/College	Sexual interest not recorded	EyeLink SR Research Remote infrared eye-tracker (500/1000 Hz sample rate recording every 2 ms)	Images—Line drawings	Partial nudity (obscured sexual regions)	10,000	2
41	Rieger et al. (2015)	USA	30	24.5	University/College/Community	Self-report (Kinsey Scale)	EyeLink SR Research Remote infrared eye-tracker (500/1000 Hz sample rate recording every 2 ms)	Video footage	Complete nudity	18,000	3

Sample: Gay men											
Study No.	Study	Country	*N*	Age	Setting	Sexual interest measure	Pupil response measure	Stimuli	Sexual explicitness	Duration per trial (ms)	Number of trials per category
4	Snowden et al. (2019)	UK	19	27.1	University/College/Community	Self-report (Kinsey Scale)	Tobii X2-60 Hz mobile eye-tracker (60 Hz rate recording every 16.67 ms)	Images—Photographs	Mixed nudity	2000	8
7	Watts et al. (2018)	UK	6	25	University/College/Community	Self-report (Kinsey Scale)	EyeLink SR Research Remote infrared eye-tracker (500/1000 Hz sample rate recording every 2 ms)	Video footage	Complete nudity	18,000	3
9	Hess et al. (1965)	USA	5	29	University/College/Community	Self-report	Manual frame-by-frame measurement of pupil diameter from video footage	Images—Photographs and paintings	Mixed nudity	10,000	5
15	Scott et al. (1967)	USA	5	26.5	Prison	Self-report	Manual frame-by-frame measurement of pupil diameter from video footage (2 f/s)	Images	Mixed nudity	10,000	8
17	Attard-Johnson et al. (2016)	UK	20	24.5	University/College	Self-report	EyeLink SR Research Remote infrared eye-tracker (500/1000 Hz sample rate recording every 2 ms)	Images—Photographs	Partial nudity (obscured sexual regions)	10,000	18
24	Rieger et al. (2013)	UK	107	23.28	University/College/Community	Self-report (Kinsey Scale)	EyeLink SR Research Remote infrared eye-tracker (500/1000 Hz sample rate recording every 2 ms)	Video footage	Complete nudity	30,000	1
41	Rieger et al. (2015)	USA	29	23.66	University/College/Community	Self-report (Kinsey Scale)	EyeLink SR Research Remote infrared eye-tracker (500/1000 Hz sample rate recording every 2 ms)	Video footage	Complete nudity	18,000	3

Sample: Bisexual men											
Study No.	Study	Country	*N*	Age	Setting	Sexual interest measure	Pupil response measure	Stimuli	Sexual explicitness	Duration per trial (ms)	Number of trials per category
4	Snowden et al. (2019)	UK	13	26.9	University/College/Community	Self-report (Kinsey Scale)	Tobii X2-60 Hz mobile eye-tracker (60 Hz rate recording every 16.67 ms)	Images—Photographs	Mixed nudity	2000	8
17	Attard-Johnson et al. (2016)	UK	19	21.1	University/College	Self-report	EyeLink SR Research Remote infrared eye-tracker (500/1000 Hz sample rate recording every 2 ms)	Images—Photographs	Partial nudity (obscured sexual regions)	10,000	18
24	Rieger et al. (2013)	UK	76	26.14	University/College/Community	Self-report (Kinsey Scale)	EyeLink SR Research Remote infrared eye-tracker (500/1000 Hz sample rate recording every 2 ms)	Video footage	Complete nudity	30,000	1
41	Rieger et al. (2015)	USA	16	24.67	University/College/Community	Self-report (Kinsey Scale)	EyeLink SR Research Remote infrared eye-tracker (500/1000 Hz sample rate recording every 2 ms)	Video footage	Complete nudity	18,000	3

Sample: Heterosexual women											
Study No.	Study	Country	*N*	Age	Setting	Sexual interest measure	Pupil response measure	Stimuli	Sexual explicitness	Duration per trial (ms)	Number of trials per category
1	Attard-Johnson et al. (2016)	UK	22	21.8	University/College	Self-report	EyeLink SR Research Remote infrared eye-tracker (500/1000 Hz sample rate recording every 2 ms)	Images—Photographs	Partial nudity (obscured sexual regions)	5000	5
2	Attard-Johnson et al. (2016)	UK	20	19.5	University/College	Self-report	EyeLink SR Research Remote infrared eye-tracker (500/1000 Hz sample rate recording every 2 ms)	Images—Photographs	Partial nudity (obscured sexual regions)	5000	5
4	Snowden et al. (2019)	UK	28	23.1	University/College/Community	Self-report (Kinsey Scale)	Tobii X2-60 Hz mobile eye-tracker (60 Hz rate recording every 16.67 ms)	Images—Photographs	Mixed nudity	2000	8
5	Snowden et al. (2019)	UK	20	21.6	University/College/Community	Self-report (Kinsey Scale)	Tobii X2-60 Hz mobile eye-tracker (60 Hz rate recording every 16.67 ms)	Images—Photographs	Mixed nudity	3000	10
7	Watts et al. (2018)	UK	9	26.2	University/College/Community	Self-report (Kinsey Scale)	EyeLink SR Research Remote infrared eye-tracker (500/1000 Hz sample rate recording every 2 ms)	Video footage	Complete nudity	18,000	3
10	Finke et al. (2018)	Germany	18	24.1	University/College	Self-report	SMI iView-X HiSpeed 500 eye-tracker (500 Hz sample rate)	Images—Photographs	Complete nudity	2500	15
11	Finke et al. (2017)	Germany	14	25.3	University/College	Self-report	SMI iView-X HiSpeed 500 eye-tracker (500 Hz sample rate)	Images—Photographs	Mixed nudity	2500	48
14	Scott et al. (1967)	USA	10	–	University/College	Sexual interest not recorded	Manual frame-by-frame measurement of pupil diameter from video footage (2 f/s)	Images—Photographs	Mixed nudity	10,000	8
19	Hamel (1974)	USA	22	20	University/College	Sexual interest not recorded	Pupillometer system model V-1165-1R	Images	Mixed nudity	5,000	8
23	Laeng & Falkenberg (2007)	Norway	14	22.5	University/College	Other	SMI iView-X HiSpeed 500 eye-tracker (500 Hz sample rate)	Images—Photographs	No nudity (face only)	10,000	10 male; 5 female
27	Rieger et al. (2016)	UK	131	21.6	University/College/Community	Self-report (Kinsey Scale)	EyeLink SR Research Remote infrared eye-tracker (500/1000 Hz sample rate recording every 2 ms)	Video footage	Complete nudity	30,000 & 180,000	12 & 3
29	Attard-Johnson et al. (2017)	UK	25	22.4	University/College	Self-report (Kinsey Scale)	EyeLink SR Research Remote infrared eye-tracker (500/1000 Hz sample rate recording every 2 ms)	Images—Photographs	Mixed nudity	2669 (average duration)	18
39	De Winter et al. (2021)	Netherlands	53	23.2	University/College	Sexual interest not recorded	EyeLink SR Research Remote infrared eye-tracker (500/1000 Hz sample rate recording every 2 ms)	Images—Photographs	Partial nudity (obscured sexual regions)	10,000	2
40	De Winter et al. (2021)	Netherlands	45	23.3	University/College	Sexual interest not recorded	EyeLink SR Research Remote infrared eye-tracker (500/1000 Hz sample rate recording every 2 ms)	Images—Line drawings	Partial nudity (obscured sexual regions)	10,000	2
19	Hamel (1974)	USA	22	20	University/College	Sexual interest not recorded	Pupillometer system model V-1165-1R	Images	Mixed nudity	5,000	8
23	Laeng and Falkenberg (2007)	Norway	14	22.5	University/College	Other	SMI iView-X HiSpeed 500 eye-tracker (500 Hz sample rate)	Images—Photographs	No nudity (face only)	10,000	10 male; 5 female
27	Rieger et al. (2016)	UK	131	21.6	University/College/Community	Self-report (Kinsey Scale)	EyeLink SR Research Remote infrared eye-tracker (500/1000 Hz sample rate recording every 2 ms)	Video footage	Complete nudity	30,000 & 180,000	12 & 3
29	Attard-Johnson et al. (2017)	UK	25	22.4	University/College	Self-report (Kinsey Scale)	EyeLink SR Research Remote infrared eye-tracker (500/1000 Hz sample rate recording every 2 ms)	Images—Photographs	Mixed nudity	2669 (average duration)	18
39	De Winter et al. (2021)	Netherlands	53	23.2	University/College	Sexual interest not recorded	EyeLink SR Research Remote infrared eye-tracker (500/1000 Hz sample rate recording every 2 ms)	Images—Photographs	Partial nudity (obscured sexual regions)	10,000	2
40	De Winter et al. (2021)	Netherlands	45	23.3	University/College	Sexual interest not recorded	EyeLink SR Research Remote infrared eye-tracker (500/1000 Hz sample rate recording every 2 ms)	Images—Line drawings	Partial nudity (obscured sexual regions)	10,000	2

Sample: Lesbian women											
Study No.	Study	Country	*N*	Age	Setting	Sexual interest measure	Pupil response measure	Stimuli	Sexual explicitness	Duration per trial (ms)	Number of trials per category
4	Snowden et al. (2019)	UK	17	26.1	University/College/Community	Self-report (Kinsey Scale)	Tobii X2-60 Hz mobile eye-tracker (60 Hz rate recording every 16.67 ms)	Images—Photographs	Mixed nudity	2000	8
7	Watts et al. (2018)	UK	9	26.2	University/College/Community	Self-report (Kinsey Scale)	EyeLink SR Research Remote infrared eye-tracker (500/1000 Hz sample rate recording every 2 ms)	Video footage	Complete nudity	18,000	3
27	Rieger et al. (2016)	UK	106	24.97	University/College/Community	Self-report (Kinsey Scale)	EyeLink SR Research Remote infrared eye-tracker (500/1000 Hz sample rate recording every 2 ms)	Video footage	Complete nudity	30,000 & 180,000	12 & 3

Sample: Bisexual women											
Study No.	Study	Country	*N*	Age	Setting	Sexual interest measure	Pupil response measure	Stimuli	Sexual explicitness	Duration per trial (ms)	Number of TRIALS per category
4	Snowden et al. (2019)	UK	21	24.3	University/College/Community	Self-report (Kinsey Scale)	Tobii X2-60 Hz mobile eye-tracker (60 Hz rate recording every 16.67 ms)	Images—Photographs	Mixed nudity	2000	8
27	Rieger et al. (2016)	UK	108	22.9	University/College/Community	Self-report (Kinsey Scale)	EyeLink SR Research Remote infrared eye-tracker (500/1000 Hz sample rate recording every 2 ms)	Video footage	Complete nudity	30,000 & 180,000	12 & 3

### Samples

Of the 19 eligible studies, 17 were classified as published, and 2 (studies 39 and 40) were not published at the time the meta-analysis was conducted but had been submitted for publication. The year the studies were conducted ranged from 1965 to 2020 and samples were drawn from the UK (*k* = 9), the U.S. (*k* = 5), Germany (*k* = 2), the Netherlands (*k* = 2), and Norway (*k* = 1). Of those studies, 18 (95%) indicated that the participants were from university, college, or community and the participants of one study were drawn from a prison population.

### Coding Procedure

All studies were coded separately by the first and second author using a coding manual (see online supplement). On completion of the independent coding, consensus was reached following any differences between the raters. To assess interrater reliability for categorical variables (*n* = 8), Cohen’s kappa (*k*) was calculated and ranged from 0.64 to 1.00 (median = 0.82) for the following variables: sexual interest measure, location type, pupil response measure, stimulus mode, stimulus explicitness, stimulus body region, stimulus presentation duration, and pupil response analysis methods. To calculate interrater reliability for the continuous variables, intraclass correlation coefficients for absolute agreements (ICC) were performed. These ranged from 0.66 to 1.00 (median = 1.00) for the following variables: sample size, mean sample age, mean SD for sample age, mean pupil response, mean SD for pupil response, correlations, and *t*-values.

### Effect Size Calculation

The effect size was calculated in Cohen’s *d* for within-subject designs using formulas 12.19–12.21 from Borenstein (2009). Cohen’s *d* measured the average difference in pupil size during the viewing of images of men and women. Separate meta-analyses were conducted for the different gender and sexual orientation groups (i.e., heterosexual men, bisexual men, gay men, heterosexual women, and lesbian women). No analysis of bisexual females was possible due to the insufficient number of studies (*k* = 2). Positive effect sizes indicate larger pupil size for same-sex stimuli, whereas negative effect sizes indicate larger pupil size for other-sex stimuli. Cohen’s *d* values of 0.20 reflect “small,” 0.50 “moderate,” and 0.90 “large” effects (Cohen, 1988). For comparison, examples of typical effect sizes for genital arousal measures while viewing male versus female sexual erotica are *d* = 1.03 for heterosexual men (Jabbour et al., 2020) and *d* = 3.20 for gay men (Semon et al., 2017), and *d* = 0.41 for Viewing Time measures in heterosexual and gay men (Ó Ciardha et al., 2018). An effect size for women of all sexual orientations may not provide a useful comparison since the direction of genital response patterns are varied across studies (see, for example, Chivers et al., 2004; Chivers, 2005; Rieger et al., 2015). However, an example of an effect size measuring genital arousal in heterosexual women is *d* = 0.64 with greater arousal to same-sex stimuli (Chivers et al., 2004) and *d* = 0.22 with no difference between male and female stimuli for Viewing Time measures (Xu et al., 2016).

All effect sizes where the 95% CI excludes 0 are equivalent to being statistically significant at *p* < 0.05. When confidence intervals for two variables do not overlap, these can be considered as being statistically different from each other, *p* < 0.01.

### Meta-Analysis

Meta-analysis is a statistical method to aggregate weighted findings from multiple samples, presuming the aggregation of findings reflects a more accurate measure of the true effects of a particular variable of interest than individual samples. Findings across samples were aggregated using both fixed-effect and random-effects meta-analysis (Borenstein et al., 2009). Fixed-effect meta-analysis assumes that all studies are estimating the same underlying effect and any variability in this estimate is due to sampling error alone (Welton et al., 2012). Random-effects meta-analysis, on the other hand, relaxes this assumption by allowing for variability in the true effect size between studies. In this model, each study has its own unique underlying effect, which in turn is generated from a probability distribution of effect sizes. Thus, the unique underlying effects for studies can be informally thought of as random samples from a distribution of effect sizes (Welton et al., 2012).

When variability across samples is low (*Q* < degrees of freedom), fixed-effect and random-effects meta-analyses produce identical results. While both fixed-effect and random-effects results are presented, we focused on the fixed-effect model for interpretation as all the results are based on a small number of samples (*k* < 30) and the between-study variability component necessary for random-effects analyses (i.e., *T*^2^, tau) becomes imprecise as the number of samples decreases (Schulze, 2007).

We used Cochran’s *Q* and *I*^2^ statistics to test the degree of heterogeneity across samples. Cochran’s *Q* statistic tests whether variability across samples in an effect size is larger than would be expected by sampling error. The *I*^2^ value is akin to a measure of effect size variability across samples and indicates the variability due to true heterogeneity, above and beyond what is expected by sampling error as opposed to chance. The *I*^2^ statistic can be compared across analyses, whereas *Q* cannot when the number of samples (*k*) varies across analyses. As a general heuristic, *I*^2^ values of 25% are considered low, 50% moderate, and 75% high variability (Higgins et al., 2003).

Meta-analyses can be strongly influenced by unusually small variance, typically due to unusually large sample sizes in between-subject *d* meta-analysis, but in within-subject meta-analyses such as the current study, by unusually large correlations estimates. Particularly in moderator analyses (when *k* decreases), the effects of other studies can largely disappear in the presence of a particularly heavy-weighted study. As in other meta-analyses (Hanson & Bussière, 1998), a study was considered an outlier and excluded if it had an extreme weight, the overall Q was significant, was the most extreme value, and it accounted for more than 50% of the overall Q. The exception to this rule was for analysis which only included three or fewer studies such that none of the studies were considered outliers. In addition to outliers, meta-analyses can also be strongly influenced by unusually large sample sizes. Therefore, to reduce the influence of an unusually heavy-weighted study, we artificially gave such a study only 10% more weight than the next largest study (see online supplement).

#### Moderator Analyses

When there was a reasonable amount of variability in an aggregated effect size (*I*^2^ > 20%) and sufficient samples reporting on the moderator of interest (2 for each level of the categorical moderator), between-level *Q* statistic analyses were conducted (Borenstein et al., 2009). A statistically significant between-level *Q* statistic analysis indicates that the moderator explains a significant portion of the variability across samples, above what would be expected by sampling error alone. Moderators examined included stimulus sexual explicitness (i.e., degree of nudity—complete nudity, partial, or dressed), pupillary measurement technique (i.e., eye tracker system used), and whether a self-report measure of sexual orientation was included.

#### Publication Bias

Publication bias occurs when studies in peer-reviewed journals have systematically different results from studies that are never published (Song et al., 2013). As such, publication bias can be a threat to meta-analysis validity if published studies are not representative of all studies that have been done on the topic (Rothstein et al., 2005). In the present study, two main publication bias tests were conducted: *P*-curves (Simonsohn et al., 2014a, 2014b, 2015) and funnel plots (Light & Pillemer, 1984; Sterne & Egger, 2001; Sterne et al., 2011). *P*-curves were generated using the “dmetar” package v. 0.0.9 (Harrer et al., 2019) in R v.3.62. (R Core Team, 2019), and funnel plot tests were conducted with the “meta” R package v. 4.10 (Balduzzi et al., 2019).

*P*-curves test whether the meta-analysis results can be explained by selective reporting practices such as *p*-hacking. A set of studies are said to have evidential value (i.e., represent a true effect) if the distribution of significant *p* values is right-skewed. This happens because smaller *p* values are more likely to occur in the presence of a true effect compared to *p* values close to 0.05. If the effect does *not* exist (and there is no *p*-hacking), the distribution of *p* values would be uniform. However, if there is *p*-hacking, the distribution would be left-skewed because there will be a disproportionate number of *p* values close to 0.05 (Simonsohn et al., 2014a).

The funnel plot is a technique where the study effect sizes are plotted against a measure of their precision, such as the standard error (Sterne & Egger, 2001). In the absence of bias, studies are expected to form a symmetrical funnel shape, where more precise studies appear narrowly at the top of the plot and less precise studies scatter more widely at the bottom. However, if the funnel shape appears asymmetric because studies are missing from one side of the plot, this could indicate the presence of publication bias (though publication bias is not the only possible cause of asymmetry; see Sterne et al., 2011).

## Results

The characteristics of the approaches and protocols used for measuring pupillary responses for the studies included in this meta-analysis is summarized in Table 2. The majority of studies confirmed sexual interests of the participants with a measure of self-report sexual orientation (*k* = 15), while four studies (De Winter et al., 2021; Hamel, 1974; Scott et al., 1967) did not record sexual orientation and instead assumed all participants were heterosexual. All included studies used images (*k* = 15) or video footage (*k* = 4) comprising either an adult male or female presented completely nude (*k* = 5), partially nude (i.e., sexual regions only obscured with clothing or image manipulation; *k* = 5), dressed (*k* = 1), or mixed stimulus sets depicting people in a range of explicitness (i.e., a mixture of nude, partially nude, and dressed images; *k* = 8). The number of trials per category ranged from 1 to 48 (median = 8), and the stimulus duration ranged from 2 s to 3 min (median = 10 s). The majority of studies conducted since the year 2000 measured pupillary responses using eye-tracking technology, such as the SR EyeLink 1000 or SMI. However, many of the earlier studies measured pupil size manually, typically by recording video footage of the eyes of observers, while these viewed stimulus images, and then manually measuring pupil size in image stills taken from this video footage (Hess et al., 1965).

**Table 2:** Meta-analysis results of pupil dilation as a measure of sexual interest

Group	Fixed-effect		Random-effects		*Q*	*I*^2^ (%)	*k*	*N*
	*d*	95% CI	*d*	95% CI				
Heterosexual men	− .26	[− 0.35, − 0.18]	− .55	[− 0.80, − 0.29]	105.90*	86.8	15	550
Gay men	.26	[0.13, 0.38]	.28	[ 0.11, 0.44]	6.68	40.14	5	65
Bisexual men	.91	[ 0.65, 1.16]	.47	[ − 0.43, 1.37]	31.11*	90.36	4	124
Heterosexual women	− .06	[− 0.15, 0.03]	− .07	[− 0.31, 0.16]	73.33*	83.64	13	403
Lesbian women	− 1.01	[− 1.30, − 0.72]	− .58	[− 3.74, 2.58]	205.44*	99.03	3	132
Bisexual women	–	–	–	–	–	–	–	–

Heterosexual men analysis included studies 1, 2, 5, 7, 9, 10, 11, 14, 15, 17, 24, 29, 39, 40, 41; gay men included studies 7, 9, 15, 17, 41; bisexual men included studies 4, 17, 24, 41; heterosexual women included studies 1, 2, 5, 7, 10, 11, 14, 19, 23, 27, 29, 39, 40; lesbian women included studies 4, 7, 27; there were insufficient bisexual women studies for analysis

*d*: effect size in Cohen’s *d*; CI: confidence interval; *Q*: *χ*^2^ test statistic of heterogeneity; *I*^2^: percentage of variability between studies that can be attributed to heterogeneity rather than sampling error. *k*: number of studies included in the analysis. *N*: total number of participants on which the analysis is based

^*^Statistically significant

The results from the meta-analysis are also presented in Table 2 and visualized in Fig. 1. The fixed-effect meta-analysis for heterosexual men yielded a small negative effect (*d* = − 0.26, fixed-effect model), indicating that they exhibited greater pupil dilation to other-sex (i.e., female) compared to same-sex (i.e., male) stimuli.^1^ There was significant variability in the studies (*I*^2^ = 87%), however, above and beyond what would be expected based on sampling error alone.

In contrast to heterosexual men, gay men showed greater pupil dilation to same-sex stimuli compared to other-sex stimuli. The effect size was similar in magnitude (*d* = 0.26 in the fixed-effect model and *d* = 0.28 in the random-effects model) and was statistically significant as the 95% CI again excluded 0. Additionally, the effects were relatively consistent across studies (*I*^2^ = 40%). Therefore, in line with their reported sexual orientation, gay men showed stronger pupil dilation to male stimuli.

The results for bisexual men yielded a similar pattern to that of gay men. Bisexual men also showed greater pupil dilation to male stimuli, but the effect size was considerably larger than that of gay men (*d* = 0.91). There was large variability between studies (*I*^2^ = 90%).

The results for heterosexual women showed a very small, statistically non-significant, negative effect size (*d* = − 0.06 in the fixed-effect and *d* = − 0.07 in the random-effects meta-analysis), indicating slightly greater pupil dilation to other-sex (male) stimuli. Therefore, while the effect was in the expected direction, its magnitude was modest. The effect did not reach statistical significance as the 95% CIs included 0. There was large variability between studies (*I*^2^ = 83%).

Finally, lesbian women showed greater pupil dilation to other-sex (male) stimuli (*d* = − 1.01). The meta-analysis estimate was not significant as the 95% CIs included 0 and variability between studies was high (*I*^2^ = 99%). This result is contrary to predictions as lesbian women were expected to exhibit greater pupil dilation to same-sex stimuli. However, this result needs to be interpreted with caution, as the effect size estimate was likely strongly influenced by one of the three included studies (No. 4; Snowden et al., 2019, Exp.1), which had a very large negative effect size (*d* = − 3.35; see Fig. 1e). Even though study No. 4 had a smaller sample size (*N* = 17) compared to study No. 27 (Rieger et al., 2016, Exp.2; *N* = 106), the weight of the two studies in the meta-analysis was almost the same (45.3% vs 44.4% in the fixed-effect model, respectively). This was because study No. 4 had smaller standard deviations compared to study No. 27, which compensated for the smaller sample size in the variance calculation of the effect size. Thus, the combination of similar weights, the overall small number of available studies in the meta-analysis, and the large (negative) effect size of study No. 4 likely contributed to the pooled meta-analysis effect being negative. No analysis of bisexual females was possible due to the insufficient number of studies (*k* = 2).^2^

### Moderator Analyses

The results of the moderator analysis are presented in Table 3 for heterosexual men and in Table 4 for heterosexual women. These analyses tested whether the effect sizes are moderated by: (1) how pupil size was measured; (2) whether a self-report measure of sexual interest was administered or not; (3) whether the correlation between group means was reported or estimated from other studies; and (4) whether participants were exposed to a high, low, or a mixed level of nudity.

**Table 3:** Moderator analyses for heterosexual men

	Fixed-effect		Random-effects		*Q*	*I*^2^ (%)	*k*	*N*
	*d*	95% CI	*d*	95% CI				
*Pupil measurement*	− **.26**	[− 0.35, − 0.18]	− **.55**	[− 0.80, − 0.29]	105.90*	86.8	15	550
Manual	− .07	[− 0.23, 0.09]	− .18	[− 0.53, 0.18]	8.44*	76.19	3	20
EyeLink	− **.68**	[− 0.81, − 0.54]	− **.85**	[− 1.20, − 0.50]	36.69*	78.2	9	481
SMI	− .11	[− 0.30, 0.08]	− .14	[− 0.41, 0.14]	1.77	43.54	2	31
Tobii	–	–	–	–	–	–	–	–
*Q*_between_					55.25*			
*Sexual interest measure*	− **.26**	[− 0.35, − 0.18]	− **.55**	[− 0.80, − 0.29]	105.90*	86.8	15	550
Not recorded ^a^	− **.36**	[− 0.49, − 0.22]	− .37	[− 0.87, 0.13]	28.42*	92.96	3	241
Self-report	− **.21**	[− 0.31, − 0.10]	− **.62**	[− 0.94, − 0.31]	74.53*	85.24	12	309
*Q*_between_					2.95			
*Correlation estimation*	− **.26**	[− 0.35, − 0.18]	− **.55**	[− 0.80, − 0.29]	105.90*	86.8	15	550
Estimated	.07	[− 0.06, 0.20]	.09	[0.00, 0.18]	1.07	0	3	33
Actual	− **.50**	[− 0.61, − 0.39]	− **.75**	[− 1.04, − 0.46]	61.91*	82.23	12	517
*Q*_between_					42.92*			
*Stimuli*^b^	− **.26**	[− 0.35, − 0.18]	− **.55**	[− 0.80, − 0.29]	105.90*	86.8	15	550
High explicitness	− **.27**	[− 0.45, − 0.10]	− **.63**	[− 1.22, − 0.05]	21.29*	85.91	4	147
Low explicitness	− **.66**	[− 0.82, − 0.51]	− **1.01**	[− 1.53, − 0.50]	27.97*	85.7	5	331
Mixed	− .03	[− 0.15, 0.08]	− .15	[− 0.39, 0.09]	17.05*	70.68	6	72
*Q*_between_					39.59*			

No eligible studies with non-nude stimuli. *d*: effect size in Cohen’s *d*; CI: confidence interval; *Q*: *χ*^2^ test statistic of heterogeneity; *I*^2^: percentage of variability between studies that can be attributed to heterogeneity rather than sampling error. *k*: number of studies included in the analysis. *N*: total number of participants on which the analysis is based

^*^Statistically significant

^a^Includes studies where sexual interest was not reported and/or not recorded. Participants were assumed heterosexual

^b^High explicitness = completely nude, low explicitness = partially nude, and mixed = a mixture of high and low or not specified

**Table 4:** Moderator analyses for heterosexual women

	Fixed-effect		Random-effects		*Q*	*I*^2^ (%)	*k*	*N*
	*d*	95% CI	*d*	95% CI				
*Pupil measurement*	− .06	[− 0.15, 0.03]	− .07	[− 0.31, 0.16]	73.33*	83.64	13	403
Manual	− .14	[− 0.31, 0.04]	− .13	[− 0.32, 0.05]	1.03	3.05	2	32
EyeLink	.02	[− 0.14, 0.17]	.02	[− 0.43, 0.48]	47.14*	87.27	7	305
SMI	.06	[− 0.12, 0.24]	− .18	[− 0.88, 0.53]	18.14*	88.98	3	46
Tobii	–	–	–	–	–	–	–	–
*Q*_between_					7.02*			
*Sexual interest measure*	− **.13**	[− 0.22, − 0.03]	− .13	[− 0.36, 0.10]	55.91*	80.33	12	350
Not recorded^a^	− .12	[− 0.27, 0.04]	− .12	[− 0.27, 0.04]	1.23	0	3	77
Self-report	− .13	[− 0.25, 0.01]	− .14	[− 0.48, 0.20]	54.66*	85.67	9	273
*Q*_between_					0.02			
*Correlation estimation*	.02	[− 0.08, 0.11]	.06	[− 0.14, 0.26]	39.89*	74.93	11	258
Estimated	− **.19**	[− 0.33, − 0.05]	− **.22**	[− 0.34, − 0.10]	2.03	1.32	3	52
Actual	**.18**	[0.06, 0.31]	.19	[− 0.06, 0.44]	22.38*	68.72	8	206
*Q*_between_					15.48*			
*Stimuli*^b^	− .05	[− 0.15, 0.04]	− .04	[− 0.27, 0.20]	56.67*	82.35	11	375
High explicitness	− .10	[− 0.27, 0.07]	− .26	[− 0.86, 0.34]	20.94*	90.45	3	158
Low explicitness	**.35**	[0.16, 0.55]	**.42**	[0.03, 0.81]	9.09*	66.99	4	140
Mixed	− **.22**	[− 0.35, − 0.08]	− **.23**	[− 0.38, − 0.07]	4.17	27.99	4	77
*Q*_between_					22.47*			

No eligible studies with non-nude stimuli. *d*: effect size in Cohen’s *d*; CI: confidence interval; *Q*: *χ*^2^ test statistic of heterogeneity; *I*^2^: percentage of variability between studies that can be attributed to heterogeneity rather than sampling error. *k*: number of studies included in the analysis. *N*: total number of participants on which the analysis is based

^*^Statistically significant

^a^Includes studies where sexual interest was not reported and/or not recorded. Participants were assumed heterosexual

^b^High explicitness = completely nude, low explicitness = partially nude, and mixed = a mixture of high and low or not specified

The effect size was significantly moderated by pupil measurement for both heterosexual men and heterosexual women. For heterosexual men, the effect was much stronger when measured with an EyeLink eye-tracker, and much weaker when measured with other equipment. In contrast, for heterosexual women, the largest effect size was obtained with manual pupil size recordings demonstrating larger pupils for other-sex stimuli.

Effect sizes were not moderated by whether the study included a self-report measure of sexual interest. This suggests that studies that reported such a measure had comparable effect sizes to those that did not report it and instead presumed that participants were heterosexual.

The type of correlation estimation also significantly moderated the effect sizes for both heterosexual men and women. For men, the effect size was stronger when the correlation could be extracted from the paper compared to when it had to be estimated using data from other similar studies. The effect sizes for heterosexual women had similar magnitude for both actual and estimated correlations. Critically, however, the effect size for estimated correlations was negative (consistent with predictions), whereas the effect size for actual correlations was positive (contrary to predictions).

Finally, the level of sexual explicitness of the visual stimuli also significantly moderated effect sizes for both heterosexual men and women. In men, the strongest effect sizes were found for low explicitness (i.e., studies with stimuli of partially nude people with obscured sexual regions), followed by high explicitness (i.e., completely nude stimuli). Mixed explicitness (i.e., stimuli containing a combination of high and low) in men led to an effect size close to 0. In contrast, the effect size for heterosexual women was positive for low explicitness (contrary to predictions) but negative for high and mixed explicitness (in line with predictions). In summary, while significant moderation was found for three out of the four considered variables, the pattern of results was generally inconsistent across heterosexual men and heterosexual women. This suggests that some of the differences in the moderator analyses may have to do, at least in part, with methodological differences in how pupil dilation is studied.

#### Publication Bias

Only the analyses for heterosexual men and heterosexual women had sufficient studies to be assessed for publication bias. The results are shown in Figure S3 in Online Supplementary Materials. Both *p*-curves were right-skewed, indicating that at least some of the studies show evidential value (i.e., represent a true effect). Therefore, the results are not likely to be explained by selective reporting based on *p* values. Note that estimating the “true” effect from *p*-curves is not recommended in cases with high heterogeneity, such as the present analyses (van Aert et al., 2016).

The funnel plots for the heterosexual male and heterosexual female analyses are presented in Figure S4 in Online Supplementary Materials. A visual investigation of the funnel plot for heterosexual women revealed no clear evidence of asymmetry. This was confirmed with Egger’s regression test (Egger et al., 1997), which showed no significant evidence for funnel plot asymmetry, *t*(11) = 0.15, *p* = 0.880. Therefore, there was no evidence that studies with heterosexual women were influenced by publication bias. In contrast, the funnel plot for heterosexual men suggested possible asymmetry, as studies were missing from the lower right side of the plot. Studies No. 1, 2, and 41 had large, negative effect sizes but also large variance, indicating lesser precision in the estimate. While it is not immediately obvious what study characteristics contributed to this, there was a lack of similarly large *positive* effect sizes with lesser precision on the left side of the funnel plot, thus leading to the apparent asymmetry. Egger’s test confirmed the significant presence of asymmetry for heterosexual men, *t*(13) = 3.53, *p* = 0.004.

The funnel plot asymmetry for heterosexual men suggests that publication bias may be present. To assess its influence on the results, we used Vevea and Woods’ (2005) bias selection model. This method uses weight functions to model the mechanisms through which effect sizes may be suppressed by publication bias and returns an adjusted estimate that corrects for this bias (Vevea & Coburn, 2019). The results from four such weight functions are presented in Table S1 (see Online Supplementary Materials). The average adjusted effect size across the four models was − 0.17 (range: − 0.15, − 0.21) for the fixed-effect meta-analysis and − 0.61 for the random-effects meta-analysis (range: − 0.48, − 0.80).^3^ Therefore, while the corrected random-effects estimate was similar to the original one, the corrected fixed-effect estimate was 65% smaller on average. In summary, the true effect size for heterosexual men may be smaller in magnitude (at least for the fixed-effect model) and this should be kept in mind when interpreting the results. However, even in the presence of such bias, the effect size was still negative, thus indicating that heterosexual men showed greater pupil dilation to female stimuli.

## Discussion

This meta-analysis examined whether changes in pupil size in response to viewing images of adult males and females can provide a consistent measure of sexual orientation in men and women. Overall, this was found to be the case for gay and heterosexual men, whereby heterosexual men showed greater pupil dilation to other-sex (i.e., female) stimuli, and gay men showed greater pupil dilation to same-sex (i.e., male) stimuli. The overall effect sizes for these observer groups ranged from small to moderate across studies, similarly to other measures of sexual interest using cognitive tasks (e.g., Viewing Time, Ó Ciardha et al., 2018), but smaller effect sizes relative to those obtained with genital arousal measurements (e.g., Jabbour et al., 2020; Semon et al., 2017). Results for bisexual men demonstrated the same direction of effect as gay men, of greater pupil dilation to male than to female stimuli. In fact, this effect was larger for bisexual than for gay men. Previous studies recording pupil dilation and genital arousal have found varied response patterns such that men who identify as bisexual inconsistently demonstrate arousal to both men and women (Rieger & Savin-Williams, 2012; Rosenthal et al., 2011). However, in some studies this group has also exhibited a stronger arousal response to men (Rieger et al., 2005; Slettevold et al., 2019; Tollison et al., 1979). These diverse findings suggest that although sexual arousal is one facet in an individual’s identification with a sexual orientation, other aspects are likely to be involved, too. One such factor is sexual curiosity. This has been found to interact with arousal responses in bisexual men, whereby those with lower levels of sexual curiosity are more aroused by same-sex men compared to those with higher level of sexual curiosity, who record bisexual arousal patterns (Rieger et al., 2013).

A different pattern was observed with female observers. Heterosexual women showed a trend of larger pupil sizes during the viewing of other-sex (i.e., male) stimuli, but the magnitude of this effect was small and not significant. The gender differences in the strength of responses observed here are consistent with those found in the wider literature on the measurement of sexual interest in men and women using other physiological measurement techniques, such as direct measures of genital arousal (Chivers et al., 2004). Similarly to the inconsistent pattern of responding in bisexual men, it is possible that sexual arousal is only one element in women’s sexual identification and may form a smaller part in the makeup of their sexual orientation than in heterosexual men (Peplau & Garnets, 2000). We return to this issue in the “Moderators of Pupil Response Measures” section.

The responses of bisexual women were not analyzed due to an insufficient number of studies (Rieger et al., 2016; Snowden et al., 2019). Lesbian women showed greater dilation to other-sex stimuli. While this last result was unexpected, the analysis included only 3 studies (total *N* = 132) and was affected by one outlier study with a large negative effect size. As the analyses of gay and bisexual groups were also based on a limited number of either 3 or 4 studies, the meta-analysis of these should be considered as preliminary until more evidence becomes available.

Finally, we note that in this study the difference scores in pupil responses for images of men and women were calculated separately for male and female observers. This provides separate response patterns and effect size estimates for men and women of different self-reported sexual orientation. This approach is applied most frequently in this literature and was taken here to examine the potential applicability of pupillary response in assessing sexual interest *within* each group. However, some studies also include comparisons between male and female observers, or between sexual orientation groups, for each image category (Rieger et al., 2012; Savin-Williams et al., 2016). These between-subject comparisons would yield different effect sizes and may also produce a different pattern of results to this meta-analysis.

### Moderators of Pupil Response Measures

We examined three moderators associated with methodological variations across studies: the sexual explicitness of stimuli, the methodological technique applied to measure pupillary response, and the inclusion of a subjective measure of sexual orientation or preference. Moderator analysis was only performed for heterosexual men and heterosexual women due to the insufficient number of studies for this analysis for the other participant groups. In heterosexual men, visual stimuli which comprised of partially nude images of people provided greater discrimination compared to studies using highly sexually explicit stimuli (i.e., complete nudity). Stimulus sets that comprised a mixture of explicitness level, such as dressed, partially nude, or completely nude, produced negligible effects. However, it is important to note that stimulus type might have been confounded with other possible moderators, such as the approach used to measure pupillary responses. Pupillary responses measured using an EyeLink eye trackers, for example, provided greater discrimination of pupil size change for male and female images in heterosexual men compared to other equipment.

These findings indicate that pupil response measurement and stimulus type can provide a source of variability. The specific effects of stimulus type and approach to measuring pupillary response are difficult to untangle because the three categories of explicitness (fully nude, partial or no nudity, and mixed nudity) do not span across the different types of pupillary response measures. For heterosexual men, for example, all studies with partially nude stimuli in the meta-analysis also applied EyeLink eye trackers to measure pupil response (Attard-Johnson et al., 2016; De Winter et al., 2021), while none of the studies using other techniques (i.e., SMI/Tobii eye trackers, manual measurement) included comparable stimuli of in low explicitness. In contrast, fully nude stimuli were employed by studies using EyeLink (Rieger et al., 2013; Watts et al., 2018) and SMI eye trackers (Finke et al., 2018), but not by any of the studies with Tobii eye trackers or manual pupil measurement. This could provide some explanation for a larger effect being observed for studies using partially nude compared to studies using more sexually explicit stimuli (i.e., complete nudity).

In heterosexual women, stimuli with low sexual explicitness demonstrated a moderate positive effect size (i.e., larger pupils when viewing persons of the same-sex), but a negative effect size (i.e., larger pupils when viewing other-sex stimuli) when mixed stimulus sets were used. The effect size for high sexual explicitness was negligible. These divergent findings could reflect the general pattern of inconsistency in female responding across studies and sexual arousal assessments more widely (Chivers, 2005; Chivers et al., 2010). Several theories have been proposed to explain inconsistencies in female sexual responding and comprehensive reviews have been published (see, for example, Chivers, 2005, 2017; Chivers et al., 2010). These theories include a range of methodological, biological, psychological and sociological factors. For example, potential differences could result from hormonal variations associated with fertility and menstrual cycle (Diamond, 2007; Shirazi et al., 2018), identification with the sexual pleasure that is perceived in other women leading to an arousal response to same-sex stimuli (Chivers, 2017), and greater malleability of sexuality by external contextual influences such as relationships (Baumeister, 2000). These possibilities have not been explored here (for a complete review of theories, see Chivers, 2017).

The comparatively small effect for stimuli of high sexual explicitness that was found in this meta-analysis could provide some support for the preparation hypothesis (Dawson et al., 2013; Lalumière et al., 2020), which suggests that any strong sexual cues provoke an indiscriminate arousal response to prepare a woman for the possibility of a sexual encounter (Chivers, 2017; Laan & Everaerd, 1995; Suschinsky & Lalumière, 2011). However, there is no clear explanation for the opposing effects of the mixed and low explicitness stimuli that were revealed in the current meta-analysis. Furthermore, the analysis considers difference scores of pupillary responses between male and female images, and from this it is therefore not possible to conclude whether the small effect is a result of no dilation *or* pupil dilation to a similar degree for both images. We also cannot exclude the possibility that other moderating factors could have contributed to this finding. For example, studies in both these subcategories included participants who were not asked to report their sexual orientation and were therefore assumed to be of heterosexual orientation (for example, De Winter et al., 2021; Hamel, 1974; Scott et al., 1967). Yet, the prevalence of same-sex behavior is reported to be higher in women compared to men (Diamond, 2016) with a difference of around 9% (7% of men versus 16% of women; Mercer et al., 2013). Thus, it is conceivable that the samples in these studies also contained a proportion of women who would have identified as bisexual or gay more so than samples of men. We note, however, that the moderator analysis also included measurement of sexual interest (i.e., whether researchers recorded self-reported sexual interest or assumed participants were heterosexual) and this was not found to moderate effect sizes.

Taken together, the findings from the present meta-analysis suggest that differences in type of stimulus and measurement technique across studies might not only influence the degree of pupillary response effects of sexual interest, but also the direction of these effects. Additional primary studies are required to draw firm conclusions on the individual influence of any of the moderators examined in the current meta-analysis.

### Limitations and Future Research Directions

Due to the low number of studies that were available, we were selective in the choices of variables that were used in the moderator analysis. Consequently, other potential variables associated with methodological techniques were not included in the meta-analysis, such as low-level stimulus characteristics. Image luminance, for example, has the potential to evoke bottom-up pupillary responses that might interfere with responses to the task-specific processes under investigation. One mechanism through which such interference could occur is the pupil light reflex, which refers to the constriction or dilation of the pupil in response to changes in the intensity of light levels that enter the eye (Ellis, 1981). The pupils begin to constrict within 200 ms after an increase in light, reaching a minimum diameter of approximately 2 mm at around 1500 ms (Mathôt, 2018). However, precise durations and pupil sizes vary depending on the intensity of the light change and individual differences (Ellis, 1981). In addition to luminance, color of the perceived light can also influence the duration of a constriction. For example, while blue light leads to sustained constriction of the pupil beyond 1500 ms, red light leads to a degree of re-dilation in the absence of light change, termed pupil escape (Mathôt, 2018). Variations in image luminance and color might, therefore, be a factor to consider when measuring pupillary responses to visual stimuli.

One method for controlling this factor is to equalize brightness across stimuli to match the mean luminance of the stimulus set (Attard-Johnson et al., 2016; Snowden et al., 2019). However, while this approach eliminates mean luminance differences across images, it could also create additional artifacts by introducing distortion of natural luminance levels *within* images, such as light–dark contrasts. Such image distortions might decrease the realism of images and could unwittingly interact with the attractiveness of a depicted person or draw the observer’s attention to information in images that is irrelevant for the task at hand. In the study of pupillary responses as an index of sexual interest, only a few studies have so far adopted such luminance manipulations, with results that are difficult to assimilate, demonstrating a need for further research (cf. Attard-Johnson et al., 2016; Snowden et al., 2019). However, most studies in this meta-analysis also demonstrate divergent pupillary responses to the same stimuli, based on differences in observers’ sex and sexual orientation, indicating that low-level image characteristics are unlikely to explain the study findings.

Another factor that was not included in the meta-analysis here is the approach that is taken to analyze pupillary response data, which also varies across research studies. A recent paper compared four widely used methods for analyzing pupil responses (Attard-Johnson et al., 2019). Specifically, Attard-Johnson et al. analyzed (unadjusted) raw pupil scores obtained from eye-tracking, *z*-scores of this data, a conversion of the pupil data that captures differences between conditions in terms of the percentage change in eye pupil size, and pupillary responses that have been adjusted on the basis of a baseline measure. They reported that fundamental pupillary response patterns were consistent across all methods.

Finally, although it was possible to obtain information on all three methodological moderators from published sources or from direct contact with the authors, we could not acquire the actual correlations for pupil responses between male and female stimuli for some of the earlier studies included in the meta-analysis. Therefore, estimated correlations were used as a proxy when coding these studies. Actual correlation data produced a larger effect size compared to estimated correlations for heterosexual men. For heterosexual women, actual and estimated correlations produced effect sizes of similar magnitude but in different directions. In order to facilitate accumulation of knowledge, studies on pupil responses should provide correlations between sets of stimuli.

### Implications for the Measurement of Sexual Interest

The current meta-analysis offers some tentative guidance for the measurement of pupillary responses of sexual interest. Across both heterosexual men and women, the low sexual explicitness stimuli (comprising partial or no nudity) appeared to provide clearer discrimination in pupillary response to sexual interest compared to high explicitness stimuli (complete nudity). We note, however, that for heterosexual women this effect was not in the direction consistent with their sexual orientation. Current evidence is too limited to determine whether some eye-tracking apparatus are better suited to this research. The desk-mounted EyeLink provided the strongest effect for heterosexual male observers, but this did not extend to heterosexual women for whom the largest effect was obtained for manual recording, and there were insufficient eligible studies for inclusion of remote Tobii eye trackers. Thus, further investigation comparing these methods is warranted, especially in women.

Furthermore, it is also possible that the affective state underlying these arousal patterns could be attributed to states evoked from other interpretations of the stimulus being perceived by the observer as pleasant or unpleasant. For instance, an arousal response would also be evoked if the stimulus is perceived as threatening or unpleasant when viewing, for example, stimuli depicting illness or violence (Bradley et al., 2008). It may not be possible to establish for certain whether sexual arousal underpins pupil dilation responses to sexual stimuli. Equally, we cannot exclude the possibility that other ‘pleasant’ emotions, for instance affection or aesthetic appeal, could be underlying the pupil dilation responses to sexual stimuli. We would argue that it is implausible that, for example, heterosexual male arousal to female images is better explained by perceived threat or unpleasantness, or pleasant emotions given that correlations of pupillary responses to sexual stimuli with genital arousal patterns, subjective sexual arousal (Rieger et al., 2015) and sexual appeal ratings (Attard-Johnson et al., 2016). These correlations provide some evidence to support the notion that pupil dilation responses to sexual stimuli observed are related to sexual arousal—at least in men. However, we acknowledge that further research is needed to untangle the affective states underlying the arousal response when viewing these images.

This highlights the importance of obtaining confirmatory measure of sexual interest to validate against pupillary responses. In many studies this was obtained through subjective self-report of sexual orientation which, at least in men, demonstrates convergence with pupillary responses (Rieger et al., 2015), cognitive tasks (Ó Ciardha et al., 2018), and other physiological measures of sexual arousal (Chivers et al., 2010; Rieger et al., 2015). Few direct attempts have been made to examine the convergent validity of pupillary responses with self-report sexual orientation and cognitive-based measures of sexual interest (Attard-Johnson et al., 2016; Ó Ciardha et al., 2018; Rieger et al., 2015) and only one study with genital responses (Rieger et al., 2015). From these, men’s pupillary responses demonstrated modest correlations with genital response (average *r* = 0.59; Rieger et al., 2015), viewing time (average *r* = 0.51; Ó Ciardha et al., 2018; Rieger & Savin-Williams, 2012), self-report sexual orientation (average *r* = 0.56; Ó Ciardha et al., 2018; Rieger et al., 2015; Rieger & Savin-Williams, 2012), and subjective sexual appeal and arousal ratings (average *r* = 0.58; Attard-Johnson et al., 2016; Rieger et al., 2015). In contrast, women’s pupillary responses correlated weakly with genital response (average *r* = 0.19; Rieger et al., 2015), subjective sexual appeal, and arousal ratings (average *r* = 0.15; Attard-Johnson et al., 2016; Rieger et al., 2015), but demonstrated a moderate correlation with viewing time tasks (average *r* = 0.47; Ó Ciardha et al., 2018; Rieger & Savin-Williams, 2012) and self-report sexual orientation (average *r* = 0.31; Ó Ciardha et al., 2018; Rieger et al., 2015; Rieger & Savin-Williams, 2012). We recommend that future primary studies seeking to use pupillary responses as a measure of sexual interest include also one or more alternative measures of sexual orientation to strengthen our understanding of the validity of pupillary responses for the assessment of sexual interests in men and women.

### Conclusion

While pupillary responses were first explored as a measure of observers’ sexual interest in others in the 1960s, there has been an increase in research in this field with modern eye-tracking equipment in recent years. These studies vary in eye-tracking methodology and the approach to data analysis, stimulus materials, procedure, and participant characteristics. They also vary in their results, justifying the need for a meta-analysis of this field. The current study demonstrates that heterosexual and gay men show pupillary responses in line with their sexual orientation, whereas bisexual men exhibit greater pupil dilation during the viewing of male versus female stimuli. There were much fewer studies sampling women. In heterosexual women, differences in pupil response to male and female stimuli were small and non-significant, whereas lesbian women displayed greater dilation to male stimuli, and there were insufficient samples for bisexual women. These results therefore indicate that pupillary responses only provide a reliable index of the sexual interests of heterosexual and gay men. Although typical effect sizes for these pupillary responses are smaller compared to genital arousal measures, this approach offers a potentially valuable tool for measuring physiological arousal in a manner that is less invasive to participants and more accessible to researchers. However, the mixed findings, especially when moderators were included, also demonstrate a clear need to increase research on women, the convergent validity of this measurement technique, and for the development of standardized testing protocols and materials.

## Supplementary Information

Below is the link to the electronic supplementary material.

Supplementary file 1 (DOCX 547 kb)

## Footnotes

1 The random-effects model yielded a larger effect size (*d* = -.55). This occurred mostly because the random-effects model tends to assign greater weight to smaller studies, which happened to have larger negative effect sizes in general (see Fig. 1a). Fixed-effect models are interpreted in the Results because random-effects require larger number of studies (closer to 30 non-overlapping samples) to estimate between study variability (tau) accurately.

2 This number reflects the number of studies with datasets that comprise a unique sample, which is a requirement for a meta-analysis. Therefore, when studies have the same sample, or overlapping samples, only one of the studies is included in the analysis.

3 The larger variability in the adjusted random-effects estimate likely occurs, at least in part, because the bias selection model also adjusts the between-study variance of the model.
