# Supplementary material for: Measurement of Sexual Interests with Pupillary Responses: A Meta-Analysis
Source: Arch Sex Behav. 2021 Sep 23;50(8):3385–411. doi: 10.1007/s10508-021-02137-y (PMC8604861; doi:10.1007/s10508-021-02137-y)
Supplement: Supplementary file 1 — (DOCX 547 kb) [file 10508_2021_2137_MOESM1_ESM.docx]

**SUPPLEMENTARY MATERIALS**

Measurement of sexual interests with pupillary responses: A meta-analytic review

2021

Janice Attard-Johnson^1,4^, Martin R. Vasilev^1^, Caoilte Ó Ciardha^2^, Markus Bindemann^2^, & Kelly Babchishin^3^

**SUPPLEMENTARY MATERIALS A - METHOD**

*Figure* S1. Illustration of flow of information through different phases of meta-analytic review


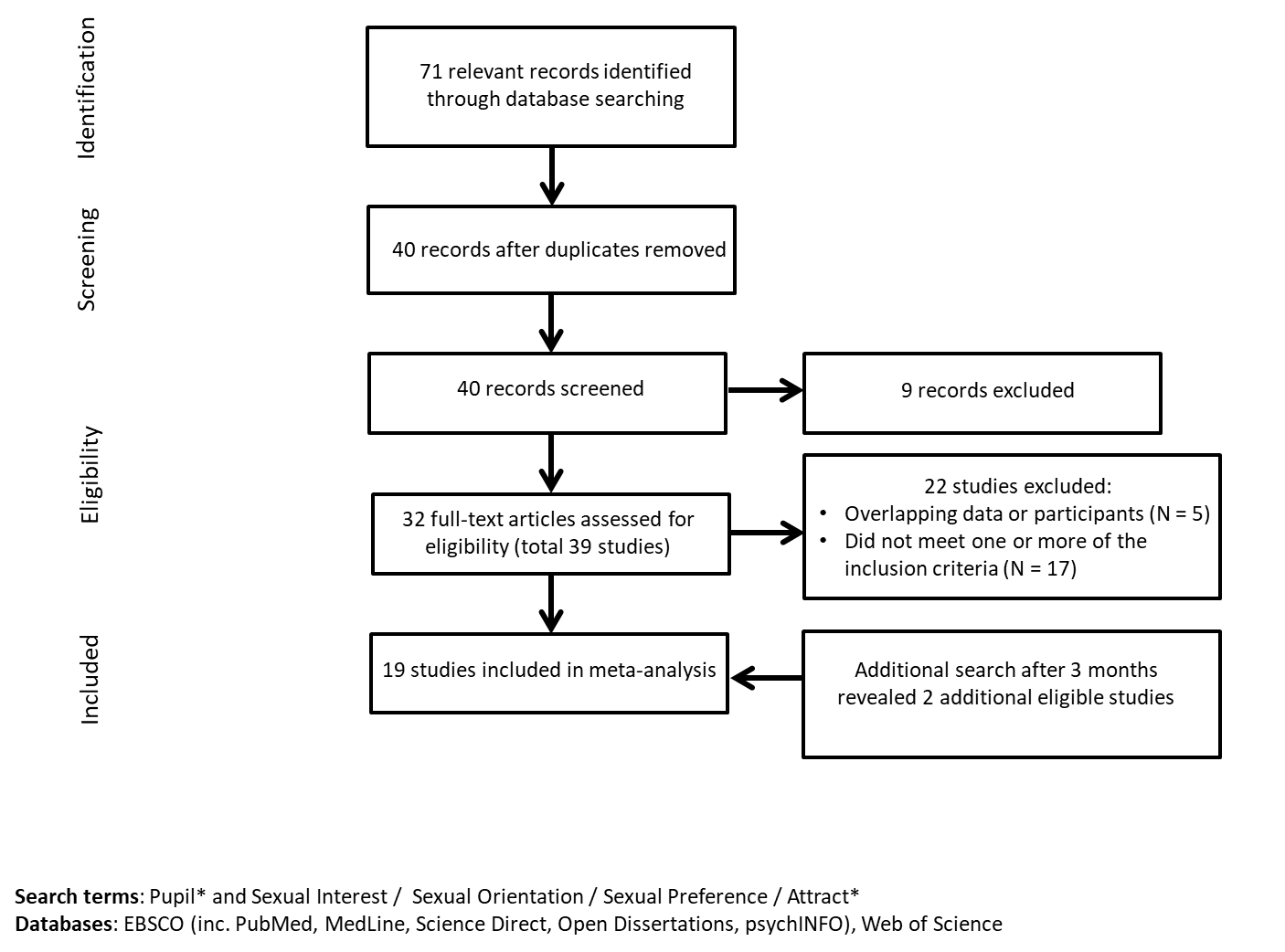


**Meta-analysis**

Extreme effect sizes can distort the pooled estimate of the meta-analysis by having an undue influence on the results. Between-study differences in a meta-analysis are often measured with a Q statistic (weighted sum of squares of the difference between the observed effect sizes and their estimate from the meta-analysis). Studies that accounted for more than 50% of the overall Q were considered an outlier and removed (Hanson & Morton-Bourgon, 2009).

In the overall Comparison 1 analysis (k = 16), study 4 was found to be an outlier and excluded as it accounted for more than 50% of the total variance (*Q*) (Hanson & Morton-Bourgon, 2009). Following removal of this study, the median value of the fixed-effect weightings (the inverse of the variance) of the individual studies (k = 15) was 21.28 and varied between 3.28 and 90.91. Five studies were identified as having unusually heavy weights (more than three times the median weight) which, in addition to outliers, can also strongly influence the meta-analysis by dwarfing the effect of studies with smaller weights. Therefore, to reduce the influence on these studies they were reweighted to 10% more than the next largest study (Study 14: 125 to 75.6; Study 10: 142.9 to 83.18; Study 5: 250 to 91.50; Studies 39 & 40: 90.91 to 68.8).

For comparison #2 (*k* = 7), Studies 4 and 24 were found to be outliers and excluded. Of the remaining studies, the fixed-effect weights varied between 6.41 and 142, with a median value of 62.5. Study 7 was reweighted from 125.0 to 68.8, and study 41 was reweighted from 142.86 to 75.68. For comparison #3 (*k* = 4) no outliers were identified. The fixed-effect weights varied between 6.8 and 250, with a median value of 16.92. Study 4 was reweighted from 250.0 to 29.7.

For comparison #4 (*k* = 14), study 4 accounted for more than 50% of the Q and was found to be an outlier and excluded. Of the remaining studies, the fixed-effect weights varied between 8.4 and 333.3, with a median value of 36.97. Studies 5 and 10 were reweighted (study 5: 166.7 to 78.6; study 10: 333.3 to 86.44). For comparison #5 (*k* = 3) there were only three studies, although study 4 accounted for more than 50% of the variance it is not recommended to consider any studies as outliers when there are only three studies as this would make the meta-analysis unstable. The fixed-effect weights varied between 4.63 and 20.41, with a median value of 20.0. As. A meta-analysis was not performed for comparison #6 (bisexual females) due to a low number of studies (*k* = 2).

To check the effect of the study exclusions above, the meta-analysis was repeated by including these studies in the analyses. Figure S2 shows the resulting forest plot. The results were generally similar, but there were also a few differences. First, the fixed effect estimate for heterosexual men changed from d= -.26 to d= 0, whereas the random-effects estimate remained similar to the results in the main text (a change from d= -.55 to d= - .43). This larger change in the fixed-effect estimate was influenced by Snowden et al.’s (2019) study, which was an outlier with a strong positive effect size (d= 2.66) and had 9.1% of the overall weight in the analysis. Because fixed-effect meta-analysis assumes that all studies are estimating the same population value and any variability is due to sampling error alone, this allowed Snowden et al.’s (2019) study to exert a larger influence on the results and pull the overall estimate towards itself. On the other hand, random-effects meta-analysis explicitly allows for between-study variability by allowing each study to have its own underlying true effect size. Therefore, it is better able to account for high heterogeneity in the individual study effect sizes. Additionally, the random-effects meta-analysis distributed the study weights more evenly compared to the fixed-effect meta-analysis, which also likely made it less susceptible to the outlier.

Second, the estimates for heterosexual women became more strongly negative, but the results did not change qualitatively (the fixed-effect estimate changed from d= -.06 to d= - .33 and the random-effects estimate changed from d= - .07 to d= - .21). Third, a similar change also occurred in the analysis of gay men, where the fixed-effect estimate changed from d= .26 to d= .89 and the random-effects estimate changed from d= .28 to d= .67. However, the pattern of results again remained the same. Finally, a new analysis of bisexual women was included, but the results should be interpreted with caution as the estimates are based on only two studies.


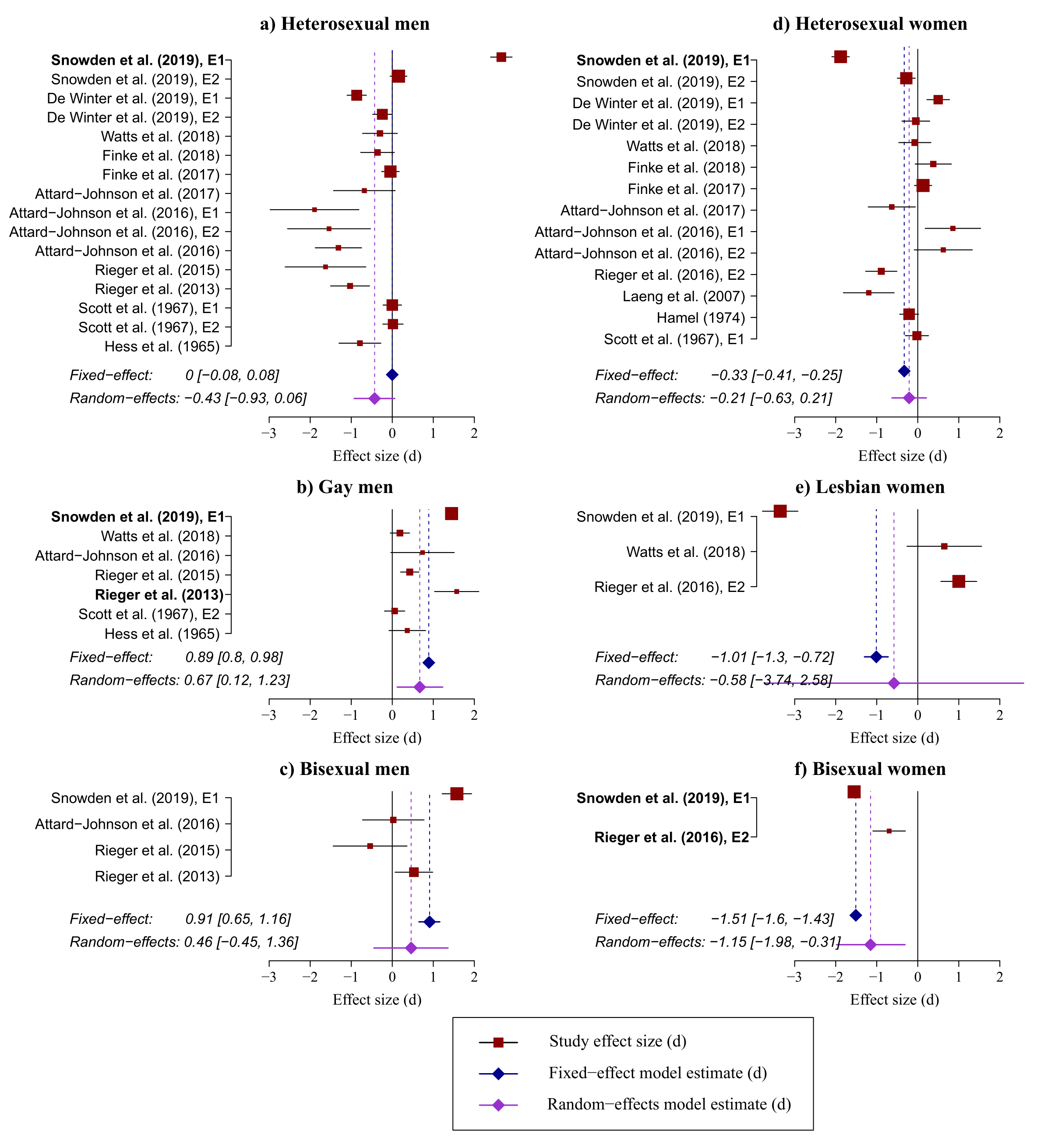


*Figure S2*. Forest plots for the main meta-analysis results for heterosexual men (**a**), gay men (**b**), bisexual men (**c**), heterosexual women (**d**), lesbian women (**e**), and bisexual women (**f**). Plotted are the effect size estimates for each study and the meta-analysis estimates (with 95% CIs). The size of squares is proportional to the fixed-effect weight of each study. Positive effect sizes indicate greater pupil dilation to same-sex stimuli and negative effect sizes indicate greater pupil dilation to other-sex stimuli. The studies which were excluded in the original forest plot have been highlighted in bold.

**SUPPLEMENTARY MATERIALS B - RESULTS**


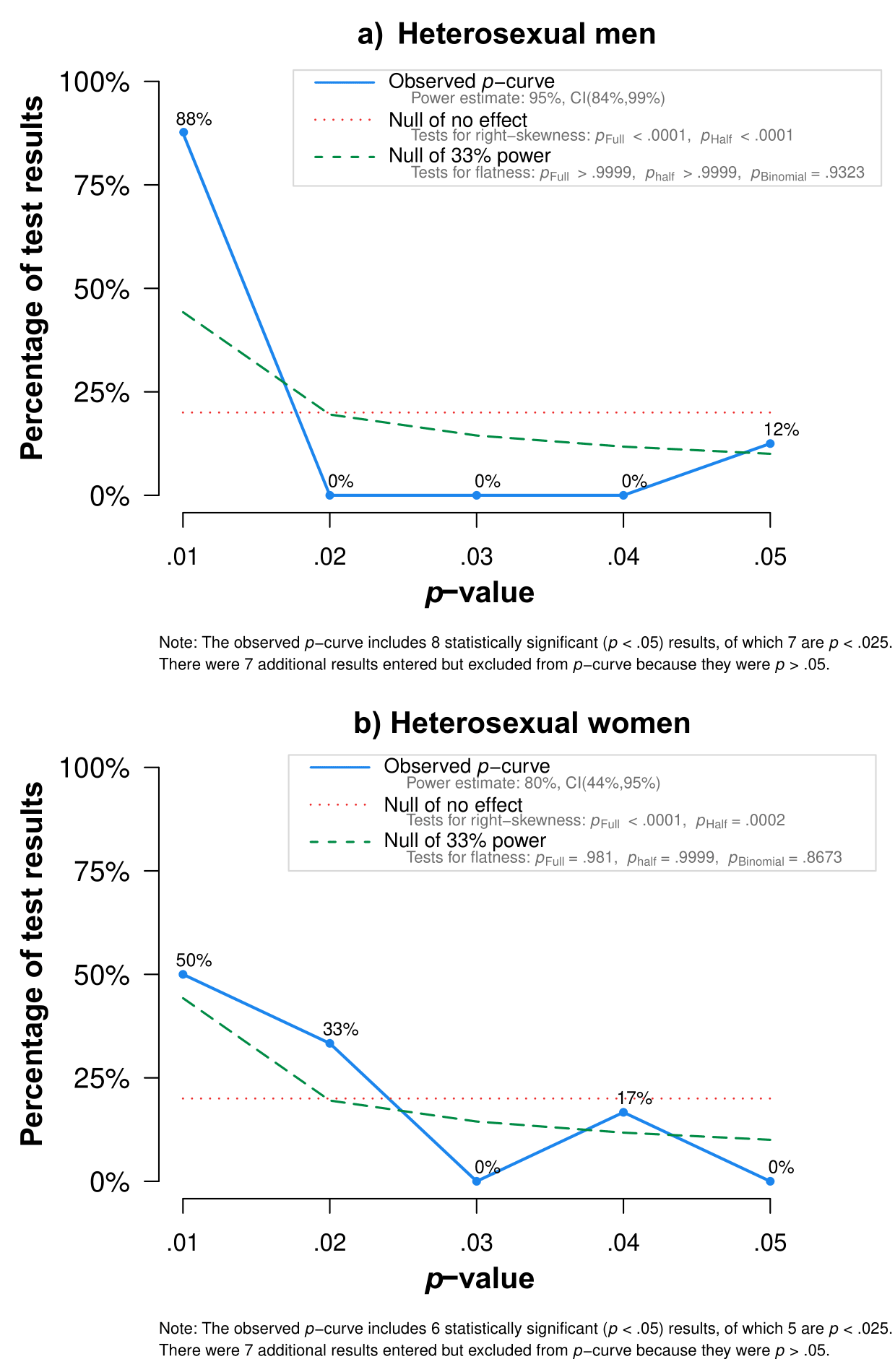


*Figure S3*. *P*-curve analysis for heterosexual men (**a**) and heterosexual women (**b**). In the absence of bias and *p*-hacking, studies are expected to form a right-skewed line. A flat curve would be expected in the absence of a true effect, and a left-skewed curve would indicate evidence for *p*-hacking (Simonsohn et al., 2014).

**
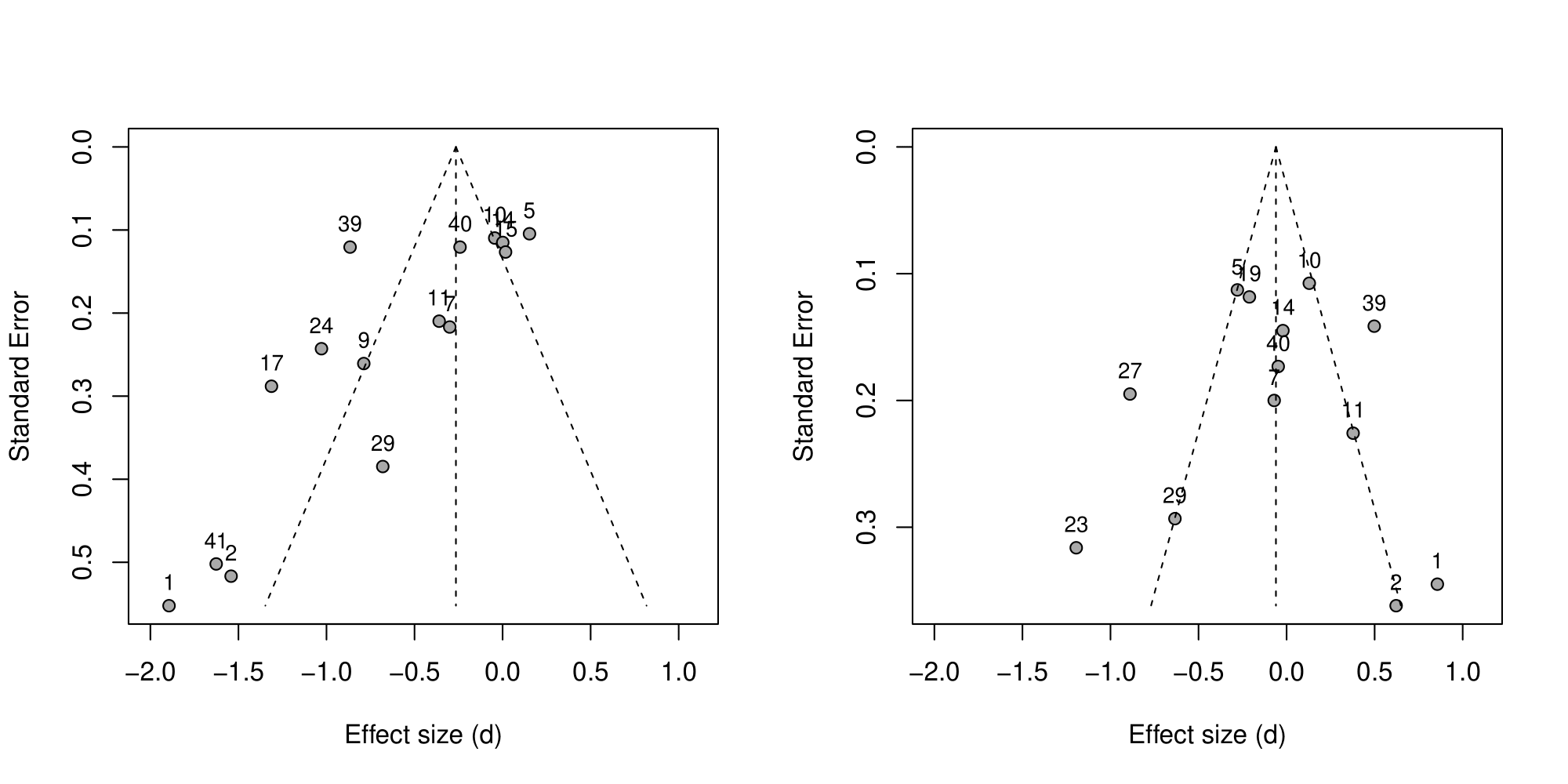
**

*Figure S4*. Funnel plots for analysis of heterosexual men (**a**) and heterosexual women (**b**). In the absence of bias, studies are expected to form a symmetrical funnel shape, with more precise studies appearing narrowly at the top of the plot and less precise studies scattering more widely at the bottom. Vertical dotted line indicates the fixed-effect meta-analysis estimate and the diagonal lines indicate 95% CIs. Numbers in the plots denote the ID number of individual studies (see Table 2).

Table S1

*Sensitivity Analysis of the Heterosexual men Meta-analysis using Vevea and Woods’ (2005) Selection Bias Model with Four Different Bias Functions*

| Analysis | Fixed-effect | Random-effects |
| --- | --- | --- |
| **Original estimate (heterosexual men)** | **-.26** | **-.55** |
| Adjusted estimate (Vevea & Woods, 2005) | | |
| Moderate one-tailed selection bias | -.18 | -.63 |
| Severe one-tailed selection bias | -.21 | -.80 |
| Moderate two-tailed selection bias | -.16 | -.53 |
| Severe two-tailed selection bias | -.15 | -.48 |
| **Mean of 4 models** | **-.17** | **-.61** |

*Note*: While Vevea and Woods’ (2005) method works with small samples such as the present one, the standard error (and therefore confidence intervals) cannot be accurately estimated. The bias functions were taken from Table 1 in Vevea and Woods (2005). The analysis was done with the “weightr” R package v.2.0.2 (Coburn & Vevea, 2019).

**SUPPLEMENTARY MATERIALS C – CODING MANUAL**

Meta Questions

Are pupillary responses to visual stimuli a robust measure for distinguishing sexual interests for male and female adults in (six separate meta-analysis based on within-subjects comparisons).

1. Straight males
2. Straight females
3. Gay males
4. Gay females
5. Bisexual males
6. Bisexual females

Based on the literature, we would expect the strongest positive effect size to be for straight males and a moderate effect size for gay males and gay females. We would expect a weaker effect size for straight females, as well as bisexual males and females.

Study eligibility:

To be included in the meta-analysis, the study had to meet the following criteria:

- Use pupillary responses to images or videos of adult men and women to measure sexual interest (adults defined as 18 and older)
- Include at least one sample of human observers that belongs to one of the following groups: straight men, straight women, gay men, gay women, bi men, bi women
- Collect measurements of pupil responses to visual content of adult men and women. This may include dressed, partially dressed, or completely nude images or video footage depicting sexually explicit or non-sexually explicit content. Images may include real photographs, artistic depictions, or computer generated images.
- Use clearly distinct stimuli (male or female images, and not mixed couples)
- Self-report sexual orientation (Kinsey scale, or other) whereby those reporting 0 and 1 (exclusively straight and mostly straight) will be categorised at heterosexual, and 5 and 6 (mostly gay and exclusively gay) will be categorised as homosexual, 2, 3 and 4 will be categorised as bisexual. Where sexual orientation information was not collected, it was taken to be heterosexual as this is the most prevalent.
- Sample size of at least 5 observers per group
- Sufficient statistical information to calculate effect size *d*.

Study Descriptive Information

One Study Identification (SI) form was completed for each study or unique sample. SI form included study identifiers as well as study descriptive statistics.

One Pupil Response form for each study/sample. The Pupil Response form included information on number of stimuli, type of stimuli, eye-tracking methodology, and method of analysis.

Study identification and description variables included:

- The year the study was published/completed.
- Whether the study is published or unpublished.
  - Published studies include peer reviewed journal articles and book chapters.
  - Unpublished include non-peer reviewed government report, web sites, thesis/dissertation, conference presentations, and unpublished manuscripts
- The largest sample size for group (each sex and orientation)
- The method sexual orientation was determined
- The average age of the sample
- The location of the sample

Pupil Response variables included:

- Number of stimuli per category
- Mode of stimuli (images, video)
- Type of stimuli
  - sexual explicitness – nude, partially nude, dressed;
  - Body exposure: Head only, full person, specific body region
- Duration of presentation of stimuli
- PR measurement method
- PR scoring method

Where information was not specified in paper, the author was contacted and information added if provided.

**Variable Coding**

Effect size

The effect size for this meta-analysis was cohen’s *d* generated from mean pupillary responses for the male and female stimuli (within-subjects). If available, the means and SDs were obtained from a table, raw data, or digitized graphs. If these were unavailable then they were obtained from test statistics or the authors were contacted. Graphs that reported standard errors or confidence intervals were converted to standard deviation.

The equation was as follows:

Within-subjects d (without r): Cohen's d_av_=M_diff_/((SD_1_+SD_2_)/2)

Within-subjects d (with r): Cohen's _drm_=( M_diff_/sqrt(SD_1_^2^+SD_2_^2^-2*r*SD_1_*SD_2_))*sqrt(2(1-r))

Variance: Vd_rm_=(1/n+d_rm_^2^/2n)2(1-r)

If there were multiple studies using overlapping samples, then the average effect size will be taken if the sample size is identical, and the effect size for the study with the largest sample will be taken if the sample size is not identical.

Dependent and Independent Variables

Our independent variable was stimulus type (male vs female adults). The dependent variable included the pupil response to these stimuli. The variables were coded separately for each observer category based on gender and sexual orientation, to conduct separate meta-analysis for each of these categories.

**CODING SHEET – PR META – 2019**

CODER (Name): ___________ DATE: ______________________

STUDY NUMBER_________________

TITLE______________________________________________________________________________________________________________________________________________________________________________________________________________________________________________________

AUTHOR(s)______________________________________________________________________________________________________________________________________________________________________________________________________________________________________________________

YEAR(S) ___________________________________________________________________________

COUNTRY___________________________________________________________________________

SOURCE_______________________________________________________________________________________________________________________________________________________________________________________________________________________________________________

NOTES____________________________________________________________________________________________________________________________________________________________________________________________________________________________________________________________________________________________________________________________________________________________________________________________________________

Inclusion Criteria:

TRUE/FALSE

| Use pupillary responses to images of men and women to measure sexual interest |  |
| --- | --- |
| Include at least one sample of human observers that belongs to one of the following groups: straight men, straight women, gay men, gay women, bi men, bi women |  |
| Collect measurements of pupil responses to visual sexual content |  |
| Use clearly distinct stimuli (male and female images, and not couples) |  |
| Self-report sexual orientation (Kinsey scale, or other). Where sexual orientation was not specified, it was taken to be heterosexual as this is the most prevalent. |  |
| Sample size of at least 5 cases per group |  |
| Sufficient statistical information to calculate effect size d. |  |

Study Included? YES/ NO

**STUDY IDENTIFICATION FORM – PR META – 2019**

CODER (Name): ___________ DATE: ______________________

- STUDY – Identification # ___________
- PUBLISHED – Yes/No
- PEERREV – Yes/No
- NMale – Total sample size of men with PR responses __________
  - N_S_Male– Total sample size of men classified as straight _______
  - N_Bi_Male– Total sample size of men classified as bisexual _______
  - N_G_Male– Total sample size of men classified as gay _______
- NFemale– Total sample size of women with PR response _________
  - N_S_Male– Total sample size of women classified as straight _______
  - N_Bi_Male– Total sample size of women classified as bisexual _______
  - N_G_Male– Total sample size of women classified as gay _______
- AMale – Mean age (SD) of men __________ (SD = ________ )
  - A_S_Male– mean age of men classified as straight _______ (SD = ____)
  - A_Bi_Male– mean age of men classified as bisexual _______(SD = ____)
  - A_G_Male– mean age of men classified as gay _______(SD = ____)
- AFemale– Mean age (SD) of women __________ (SD = ________ )
  - A_S_Female– mean age of women classified as straight _______ (SD = ____)
  - A_Bi_Female– mean age of women classified as bisexual _______(SD = ____)
  - A_G_Female– mean age of women classified as gay _______(SD = ____)
- SIMEASURE – assessment method of sexual interest
  - 1 = Self- report Kinsey scale
  - 2 = Self-report Other
  - 3 = Other ______________
  - 4 = Not Specified (Assumed heterosexual)
- LOCNTYPE _________
  - 1 = university/colleges
  - 2 = community
  - 3 = prison
  - 4 = other
  - 5 = University/College/Community

**PUPIL RESPONSE CONSTRUCTION FORM – PR META – 2019**

- PRMEASURE – pupil response measurement methods: __________
  - 1 = Manual
  - 2 = EyeLink
  - 3 = Tobii
  - 4 = SMI
  - 5 = other: ______________________________
- PRSTIMMODE – mode of stimuli used: __________
  - 1 = Image
  - 2 = Video
- PRSTIMEXP – exposure of stimulus: ______________
  - 1 = complete nudity (visible sexual regions)
  - 2 = partial nudity (obscured sexual regions e.g, swimwear or blurring)
  - 3 = no nudity (arms and legs may be uncovered)
  - 4 = not specified
  - 5 = mixed nudity
- PRSTIMREG – body region of stimulus: __________
  - 1 = head only
  - 2= full person
  - 3 = body part
  - 4 = not specified
  - 5 = mixed regions
- PRSTIMDUR – duration of presentation of stimuli (indicate duration or if variable, mean and SD) ____
  - 0 = not specified
  - 1 = specified
  - 2 = variable (dependent on participant’s response)
- PRSTIMTRIALS – number of trials per image category indicated (indicate number) ____
  - 0 = no
  - 1 = yes
- PRSCORE – measurement scoring method utilized
  - 1 = standardized (z) score
  - 2 = percentage difference
  - 3 = baselines correction
  - 4 = raw scores
  - 5 = other __________

**VARIABLE CODING – PR META 2019**

***[To be completed separately for each sex and orientation]***

Variable name: Pupil Response Study # _______

Description of how data was obtained or extracted: _______________________________________________________________________________________________________________________________________________________________________________________________________________________________________________________________________________________

Comparison for:________________

1 = straight males

2 = gay males

3 = bi males

4 = straight females

5 = gay females

6= bi females

Page # _________________ Table / Figure _______________

|  | Mean | Standard deviation | Sample Size | (space for any calculations) |
| --- | --- | --- | --- | --- |
| Female images |  |  |  |  |
| Male images |  |  |  |  |

| Correlation |  | Estimated: Y/N |
| --- | --- | --- |

Direction of result (greater dilation) = Preferred sex / Non preferred sex / Not applicable (Bisexual)

** within subjects comparisons

If other than means and SD, what statistics were used to compute *d* _________________

Order for selection of data for calculating effect size:

1. Means and SD obtained from Tables
2. Means and SD obtained from digitized graphs
3. Mean and SD obtained from RAW data reported in manuscript
4. Test statistics
5. Contact authors
6. Other: __________

**FORMULA FOR CALCULATING D_RM_**

***From sample sizes (N), means (M), and standard deviations (SD), correlation (r)***


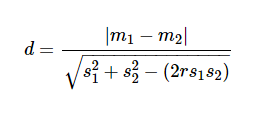


***From paired samples t-test***

d_RM_ = t / (sqrt of N)

**REFERENCES**

Coburn, K. M. & Vevea, J. L. (2019). *weightr: Estimating weight-function models for publication bias*. R package version 2.0.2. https://CRAN.R-project.org/package=weightr

Hanson, R. K., & Morton-Bourgon, K. E. (2009). The accuracy of recidivism risk assessments for sexual offenders: a meta-analysis of 118 prediction studies. *Psychological Assessment, 21,* 1-21. doi:10.1037/a0014421

Simonsohn, U., Nelson, L. D., & Simmons, J. P. (2014). P-curve: A key to the file-drawer. *Journal of Experimental Psychology: General*, *143*, 534–547. doi:10.1037/a0033242

Vevea, J. L., & Woods, C. M. (2005). Publication bias in research synthesis: Sensitivity analysis using a priori weight functions. *Psychological Methods*, *10*, 428–443. doi:10.1037/1082-989X.10.4.428
